# Causal association between type 2 diabetes mellitus and acute suppurative otitis media: insights from a univariate and multivariate Mendelian randomization study

**DOI:** 10.3389/fendo.2024.1407503

**Published:** 2024-05-21

**Authors:** Lihong Kui, Cheng Dong, Junyu Wu, Feinan Zhuo, Bin Yan, Zhewei Wang, Meiling Yang, Canhai Xiong, Peng Qiu

**Affiliations:** ^1^Xiamen Rehabilitation Hospital, Xiamen, Fujian, China; ^2^Depart of Rehabilitation Medicine, Ruijin Hospital, Shanghai Jiao Tong University School of Medicine, Shanghai, China; ^3^School of Physical Education, Shanghai University of Sport, Shanghai, China; ^4^Department of Rehabilitation, The First Affiliated Hospital of Wenzhou Medical University, Wenzhou, Zhejiang, China; ^5^School of Rehabilitation Medicine, Jiangsu Vocational College of Medicine, Jiangsu, China

**Keywords:** acute suppurative otitis media (ASOM), type 2 diabetes mellitus (T2DM), hearing loss (HL), causal relationship, Mendelian randomization (MR)

## Abstract

**Background:**

Type 2 diabetes mellitus (T2DM) and hearing loss (HL) constitute significant public health challenges worldwide. Recently, the association between T2DM and HL has aroused attention. However, possible residual confounding factors and other biases inherent to observational study designs make this association undetermined. In this study, we performed univariate and multivariable Mendelian Randomization (MR) analysis to elucidate the causal association between T2DM and common hearing disorders that lead to HL.

**Methods:**

Our study employed univariate and multivariable MR analyses, with the Inverse Variance Weighted method as the primary approach to assessing the potential causal association between T2DM and hearing disorders. We selected 164 and 9 genetic variants representing T2DM from the NHGRI-EBI and DIAGRAM consortium, respectively. Summary-level data for 10 hearing disorders were obtained from over 500,000 participants in the FinnGen consortium and MRC-IEU. Sensitivity analysis revealed no significant heterogeneity of instrumental variables or pleiotropy was detected.

**Results:**

In univariate MR analysis, genetically predicted T2DM from both sources was associated with an increased risk of acute suppurative otitis media (ASOM) (In NHGRI-EBI: OR = 1.07, 95% CI: 1.02-1.13, *P* = 0.012; In DIAGRAM: OR = 1.14, 95% CI: 1.02-1.26, *P* = 0.016). Multivariable MR analysis, adjusting for genetically predicted sleep duration, alcohol consumption, body mass index, and smoking, either individually or collectively, maintained these associations. Sensitivity analyses confirmed the robustness of the results.

**Conclusion:**

T2DM was associated with an increased risk of ASOM. Strict glycemic control is essential for the minimization of the effects of T2DM on ASOM.

## 1 Introduction

Hearing loss (HL) is a prevalent sensory impairment disease that affects approximately 430 million people around the world (1, 2). Acute otitis media (AOM) is one of the common hearing disorders facing general practitioners and otolaryngologists (3). Although AOM mostly occurs in children, the incidence rate of AOM in adults is around 5/1000 person-years (4). Acute suppurative otitis media (ASOM) is a subtype of AOM (5). ASOM often presents with fever, otalgia, ear purulence, tympanic membrane congestion, and tympanic perforation (6). Persistent ASOM can turn into chronic suppurative otitis media (CSOM) and cause HL (7). Untreated patients with CSOM may also develop further serious complications, including potentially lethal otitic meningitis and brain abscess (8, 9).

Diabetes mellitus (DM) is a metabolic disease characterized by hyperglycemia (10). Long-term hyperglycemia can lead to chronic damage and dysfunction of various organs, especially kidneys, blood vessels, nerves, and eyes (11). It is reported that the number of DM patients worldwide will increase to 783 million by 2045 as the escalating global prevalence of DM (12–14). Type 2 diabetes mellitus (T2DM) accounts for 90%-95% of DM patients. T2DM results from a complex inheritance-environment interaction along with other risk factors such as age, obesity, and physical inactivity (15–17). T2DM is characterized by hyperglycemia, insulin resistance, and insulin secretion disorders (18, 19). Patients with T2DM are always complicated with cardiovascular and cerebrovascular diseases and digestive tract dysfunction due to metabolic disorders and decreased resistance, which seriously affect the quality of life of patients (20).

Previous studies have highlighted a greater incidence of HL in DM patients compared to nondiabetic patients, and revealed a direct correlation between the severity of DM and the extent of HL (21–23). Patients with T2DM have shown both HL and vestibular dysfunction (24). It has also been shown that the insulin/glucose signaling pathology in T2DM can lead to inner ear pathology and accompanying HL (25). However, other studies suggested that the association between DM and HL does not exist when age, sex, and hypertension were taken into account (23). The causal associations between T2DM and HL remain ambiguous.

Mendelian randomization (MR) is a type of instrumental variables (IVs) analysis, which has been increasingly used in observational studies in recent years. MR uses independent single nucleotide polymorphisms (SNPs) with strong associations with exposure as IVs, facilitating to estimate of causal associations between exposure and outcomes (26). This effectively minimizes the potential residual confounding factors and other biases inherent in studies with an observational design.

In this study, we investigated the association between T2DM and 10 distinct types of HL disorders (conductive HL, sensorineural HL, mixed conductive and sensorineural HL, otitis externa (OE), otitis media (OM), ASOM, nonsuppurative OM, perforation of the tympanic membrane, hearing difficulty/problems with background noise, and sudden idiopathic HL) with T2DM. Univariate and multivariate MR analysis was used to examine the association between T2DM from two databases and 10 common types of HL disorders.

## 2 Materials and methods

### 2.1 Genetic instrumental variables selection

**Figure 1:**
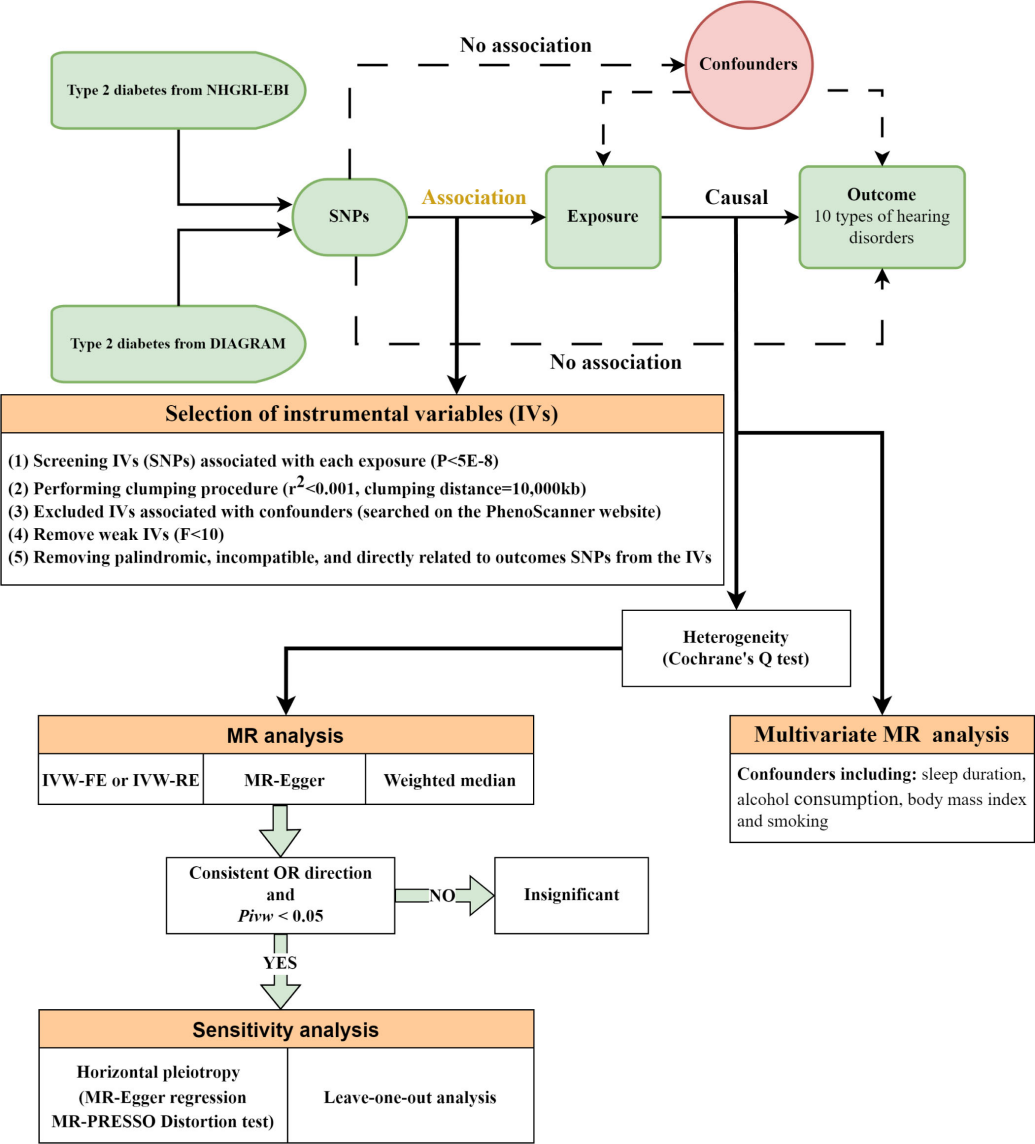
Overall design of the Mendelian randomization analysis. NHGRI-EBI, National Human Genome Research Institute-European Bioinformatics Institute; DIAGRAM, DIAbetes Genetics Replication and Meta-analysis; SNPs, single-nucleotide polymorphisms; MR, mendelian randomization; IVW, inverse-variance weighted; IVW-FE, fixed-effect IVW; IVW-RE, random-effect IVW; OR, odds ratios.

The overall design of the MR analysis in this study is shown in **Figure 1**. This MR study utilized GWAS data for T2DM in European populations, categorized according to the ICD-10 standards, from the consortium NHGRI-EBI, for first validation. The data originated from a cross-population GWAS meta-analysis of genetic associations across 220 human phenotypes (27). We further repeated verification through DIAGRAM consortium data, a GWAS study that performed a meta-analysis of genetic variants in T2DM involving nearly 150,000 people, the vast majority of whom were of European ancestry (28). More information about the exposure is presented in **Table 1**.

**Table 1:** Details of exposure and outcome GWAS datasets.

Dataset type	GWAS ID	Author	Consortium	Population	Sample size	PMID
Exposure						
Type 2 diabetes	ebi-a-GCST90018926	Sakaue S	NHGRI-EBI	European	38,841 cases and 451,248 controls	34594039
Type 2 diabetes	ieu-a-26	Morris	DIAGRAM	European	12,171 cases and 56,862 controls	22885922
Confounders						
Sleep duration	ebi-a-GCST003839	Jones SE	NHGRI-EBI	European	127,573	27494321
Alcohol consumption	ukb-b-5359	Ben Elsworth	MRC-IEU	European	64,979	NA
Body mass index	ebi-a-GCST006368	Hoffmann TJ	NHGRI-EBI	European	315,347	30108127
Smoking status	ebi-a-GCST90029014	Loh PR	NHGRI-EBI	European	468,170	29892013
Outcome						
Conductive hearing loss	finn-b-H8_HL_CON_NAS	NA	FinnGen	European	1,255 cases and 196,592 controls	NA
Sensorineural hearing loss	finn-b-H8_HL_SEN_NAS	NA	FinnGen	European	15,952 cases and 196,592 controls	NA
Mixed conductive and sensorineural hearing loss	finn-b-H8_HL_MIX_NAS	NA	FinnGen	European	1,863 cases and 196,592 controls	NA
Otitis externa	finn-b-H8_EXTOTITIS	NA	FinnGen	European	5,232 cases and 205,939 controls	NA
Otitis media	finn-b-H8_OTIMEDNAS	NA	FinnGen	European	1,832 cases and 205,939 controls	NA
Acute suppurative otitis media	finn-b-H8_SUP_ACUTE	NA	FinnGen	European	3,076 cases and 214,101 controls	NA
Nonsuppurative otitis media	finn-b-H8_NONSUPPNAS	NA	FinnGen	European	4,381 cases and 205,939 controls	NA
Perforation of tympanic membrane	finn-b-H8_PERFORATION	NA	FinnGen	European	1,469 cases and 205,939 controls	NA
Hearing difficulty/problems with background noise	ukb-b-18275	Ben Elsworth	MRC-IEU	European	171,586 cases and 281,896 controls	NA
Sudden idiopathic hearing loss	finn-b-H8_HL_IDIOP	NA	FinnGen	European	1,491 cases and 196,592 controls	NA

GWAS, genome-wide association study; NHGRI-EBI, National Human Genome Research Institute-European Bioinformatics Institute; DIAGRAM, DIAbetes Genetics Replication And Meta-analysis; MRC-IEU, Medical Research Center-Integrative Epidemiology Unit; MR, mendelian randomization; NA, not applicable.

In this MR analysis, we employed SNPs that exhibit robust associations (defined by a genome-wide significance threshold of *P*< 5×10^-8^) with T2DM as IVs (29). We meticulously selected these IVs to ensure minimal linkage disequilibrium, setting the correlation coefficient threshold to r²< 0.001 and adopted a clumping window exceeding 10,000kb to guarantee the independence of the IVs (26). To address potential confounders — namely sleep patterns, alcohol consumption, smoking, and body mass index — which could impact exposure or outcomes (30–36), we utilized the PhenoScanner tool (http://www.phenoscanner.medschl.cam.ac.uk/) to identify and subsequently exclude any SNPs linked to these confounding factors (*P*< 5×10^-8^). During the statistical harmonization process between exposure and outcome data, we meticulously removed palindromic SNPs incompatible with our analysis and SNPs directly associated with the outcomes under study. Additionally, we excluded SNPs with available exposure data but lacking corresponding outcome information. To address and minimize the potential bias from weak IVs, we calculated the F-statistic for each IV using the formula $F_{exposure}=\frac{Beta_{exposure}^{2}}{SE_{exposure}^{2}}$ (37). IVs exhibiting F-statistics less than 10 were systematically excluded from our analysis to mitigate the risk of bias associated with weak IVs (38).

### 2.2 Data sources of hearing disorders

Details of the outcomes (10 types of hearing disorders) can be found in **Table 1**. Data on nine of these hearing disorders were sourced from the FinnGen database, an extensive public-private consortium dedicated to the amalgamation and analysis of genetic and health data from approximately 500,000 participants in Finnish biobanks (39). The data on “hearing difficulty/problems with background noise” as one of the types of HL disorders came from the Medical Research Council Integrated Epidemiology Unit (MRC-IEU). Participants in this study were asked on an ACE touchscreen, “Do you find it difficult to hear conversations if there is background noise, such as from a television, radio, or children playing?” (https://biobank.ndph.ox.ac.uk/showcase/field.cgi?id=2257).

### 2.3 Data sources for confounders

Analyses were adjusted for sleep duration, alcohol consumption, body mass index, and smoking status by applying multivariate MR (MVMR). Adjustments for sleep duration were based on summary-level statistics derived from a comprehensive genome-wide association study encompassing over 120,000 individuals (40). Summary-level data for alcohol consumption were obtained from MRC-IEU (41). Summary-level data for body mass index were extracted from a large multiethnic genome-wide association study of adult body mass index to identify novel loci (42). Summary-level data for smoking status were available from a mixed-model association study for biobank-scale datasets from more than 450,000 European samples (43). More information is presented in **Table 1**.

### 2.4 Statistical analyses

In our analysis, we applied the Inverse-Variance Weighted (IVW) approach as the principal analytical method (44). The selection between fixed-effect (IVW-FE) and random-effect (IVW-RE) models was contingent on Cochrane’s Q heterogeneity test outcomes: IVW-RE was used in cases of detected heterogeneity (*P*< 0.05) to provide conservative estimates, while the IVW-FE model was employed in the absence of such heterogeneity (45).

Additionally, our analysis incorporated two supplementary MR methodologies: MR-Egger and weighted median, to bolster result credibility and ascertain causality direction. MR-Egger was used when assuming substantial horizontal pleiotropy among over half of the IVs, whereas the weighted median approach assumes less extensive pleiotropy (46, 47). Causality was inferred only when consistent effects were observed across all employed MR methods, supplemented by significant findings from IVW analysis, thus ensuring a robust and concise evaluation of the studied associations.

Furthermore, for sensitivity analysis, horizontal pleiotropy was assessed using the MR-Egger intercept’s *P*-value (46, 48). The significance of the intercept was determined by its *P*-value, which indicates whether there is a pattern of pleiotropy that could bias the causal estimates. Also, we incorporated the MR-PRESSO distortion test to check the consistency of MR estimates after excluding potential pleiotropic outliers (48). This test identified and corrected for outliers in the IVs analysis that could be attributed to pleiotropy and the subsequent recalculations, thereby ensuring the robustness of our findings against such biases. A leave-one-out sensitivity analysis was conducted to determine the impact of each individual SNP on the overall MR estimation, which involves recalculating the causal effect with one SNP removed at a time. Significant alterations in MR estimates upon SNP exclusion suggest potential biases, whereas stable results across variations indicate robust findings.

Multivariable Mendelian Randomization (MVMR) extends the conventional MR analysis to enable the simultaneous assessment of multiple exposures on an outcome, proving invaluable for adjusting potential confounders and exploring the collective impact of several exposures (49). In this study, we considered four significant confounders: sleep duration, alcohol consumption, body mass index, and smoking status.

Upon compiling GWAS summary datasets for T2DM and the aforementioned confounders, we verified that each IV maintained a strong association (*P*< 5x10^-8^) with at least one exposure or confounder. To mitigate the effects of linkage disequilibrium, SNPs were pruned within a 10,000 kb window with an r^2^ threshold of< 0.001. The IVW method was then utilized to discern the causal effects, post exclusion of palindromic SNPs and those absent in the outcome data, while accounting for the identified confounders, ensuring a refined and methodologically sound approach to our MVMR analysis.

Statistical significance was set at a threshold of *P*< 0.05. The outcomes of the causal relationships were expressed in terms of odds ratios (OR) with 95% confidence intervals (95% CI). These procedures were implemented using the “TwoSampleMR” (version 0.5.6) and “MRPRESSO” (version 1.0) (50, 51) tools in R software (version 4.2.3), ensuring a concise yet comprehensive analysis.

## 3 Results

**Figure 2:**
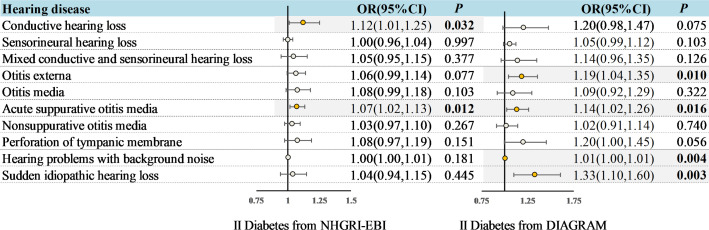
Associations between genetically predicted T2DM and hearing disorders based on the IVW approach. OR, odds ratio; CI, indicates confidence interval.

**Figure 3:**
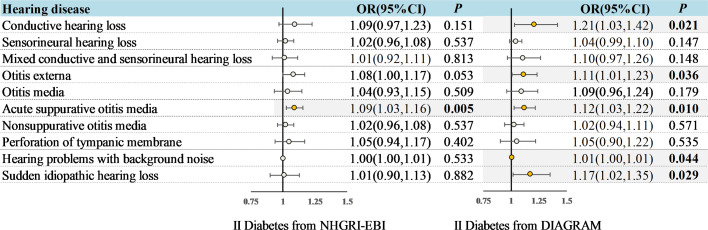
Multivariable MR analysis: Assessing the impact of T2DM on hearing disorders with adjustments for sleep, alcohol consumption, body mass index, and smoking. OR, odds ratio; CI, indicates confidence interval.

Eligible 164 and 9 SNPs were screened out from the T2DM GWAS data sets of NHGRI-EBI and DIAGRAM respectively as IVs. Selected SNPs’ characteristics were detailed in **Supplementary Table S1**, indicating minimal risk of weak instrument bias, with F statistics ranging between 29.61 and 1066.63. The confounder information was detailed in **Supplementary Table S2**. IVW results of univariable and multivariable MR analysis were shown in **Figures 2** and **3**.

### 3.1 Univariable MR analysis results

The analysis of T2DM from NHGRI-EBI has identified heterogeneity in the assessment of sensorineural HL, hearing difficulty/problems with background noise, and mixed conductive and sensorineural HL (*P_heterogeneity_* < 0.05; **Table 2**). Therefore, the IVW-RE model was used as the primary MR method for analysis in these cases. For other analyses, the IVW-FE model was employed.

**Table 2:** Results of sensitivity analyses in univariable MR analysis.

Outcome	Heterogeneity				Pleiotropy				
	MR Egger		IVW		Egger_intercept	*P*_Egger_intercept_	MR-PRESSO		
	Q	*P*_Q_	Q	*P*_Q_			*P*_Global Test_	Outliers	*P*_Distortion Test_
Type 2 diabetes from NHGRI-EBI									
Conductive hearing loss	180.51	0.152	180.53	0.165	0.001	0.910	0.170	NA	NA
Sensorineural hearing loss	204.43	0.013	207.79	0.010	0.005	0.105	0.008	rs17496664	0.085
Mixed conductive and sensorineural hearing loss	197.08	0.031	204.30	0.016	-0.018	0.016	0.012	NA	NA
Otitis externa	185.37	0.101	186.70	0.098	-0.006	0.282	0.100	NA	NA
Otitis media	174.66	0.235	175.93	0.231	-0.007	0.279	0.228	NA	NA
Acute suppurative otitis media	165.03	0.419	165.26	0.436	0.002	0.632	0.427	NA	NA
Nonsuppurative otitis media	172.78	0.267	173.08	0.280	-0.002	0.598	0.284	NA	NA
Perforation of tympanic membrane	174.88	0.231	176.33	0.225	-0.009	0.247	0.235	NA	NA
Hearing difficulty/problems with background noise	243.70	0.001	244.40	0.001	0.001	0.499	0.001	rs10195132, rs35895680	0.953
Sudden idiopathic hearing loss	186.93	0.088	190.99	0.066	0.015	0.062	0.062	NA	NA
Type 2 diabetes from DIAGRAM									
Conductive hearing loss	4.95	0.666	5.76	0.674	0.071	0.397	0.681	NA	NA
Sensorineural hearing loss	2.00	0.960	2.01	0.981	0.003	0.911	0.988	NA	NA
Mixed conductive and sensorineural hearing loss	5.43	0.607	5.53	0.700	-0.020	0.771	0.741	NA	NA
Otitis externa	11.03	0.138	11.32	0.184	-0.027	0.681	0.183	NA	NA
Otitis media	6.75	0.456	7.05	0.532	-0.036	0.602	0.529	NA	NA
Acute suppurative otitis media	7.27	0.401	7.45	0.489	0.017	0.695	0.515	NA	NA
Nonsuppurative otitis media	7.32	0.396	8.47	0.389	0.046	0.331	0.423	NA	NA
Perforation of tympanic membrane	2.02	0.959	3.62	0.890	0.092	0.247	0.908	NA	NA
Hearing difficulty/problems with background noise	12.82	0.077	13.02	0.111	0.001	0.747	0.148	NA	NA
Sudden idiopathic hearing loss	3.48	0.838	3.56	0.895	-0.021	0.785	0.919	NA	NA

NHGRI-EBI, National Human Genome Research Institute-European Bioinformatics Institute; DIAGRAM, DIAbetes Genetics Replication and Meta-analysis; IVW, inverse-variance weighted; SNP, single nucleotide polymorphism.

NA, not applicable.

Genetically predicted T2DM was associated with an increased risk of ASOM (In NHGRI-EBI: OR = 1.07, 95%CI: 1.02-1.13, *P* = 0.012; In DIAGRAM: OR = 1.14, 95%CI: 1.02-1.26, *P* = 0.016) in the univariable MR analysis (**Figure 2**). Also for genetically predicted T2DM, there was an association with an increased risk of conductive HL (OR= 1.12, 95%CI: 1.01-1.25, *P* = 0.032) as derived from the NHGRI-EBI, and an association with an increased risk of OE (OR= 1.19, 95%CI: 1.04-1.35, *P* = 0.010), hearing difficulty/problems with background noise (OR= 1.01, 95%CI: 1.00-1.01, *P* = 0.004), and sudden idiopathic HL (OR= 1.33, 95%CI: 1.10-1.60, *P* = 0.003) as derived from DIAGRAM (**Figure 2**). MR-Egger and weighted median analysis shown a consistent direction of effect (**Supplementary Table S3**-**4**). Beyond the above, the analysis shown no significant association between T2DM and other hearing disorders.

In our sensitivity analysis, we found no evidence of pleiotropy in the MR-Egger regression (*P* for Egger intercept > 0.05) and the MR-PRESSO Global Test (*P* > 0.05) when examining the impact of T2DM on ASOM, utilizing data from NHGRI-EBI and DIAGRAM (**Table 2**). Furthermore, the leave-one-out sensitivity analysis affirmed the consistency of our MR findings, indicating that no individual SNP disproportionately influenced the results (details provided in **Supplementary Figures S1**, **S2**).

### 3.2 MVMR analysis result

To deepen our understanding of the causal connections between T2DM and various hearing disorders, we conducted an additional MVMR analysis. In multivariable MR analyses adjusting for sleep duration, alcohol consumption, body mass index and smoking alone (**Supplementary Tables S5**-**8**) or together (**Figure 3**), we found only ASOM to be associated with T2DM from both databases (In NHGRI-EBI: OR = 1.09, 95%CI: 1.03-1.16, *P* = 0.005; In DIAGRAM: OR = 1.12, 95%CI: 1.03-1.22, *P* = 0.010). Notably, this association was more pronounced than those identified through univariate MR analyses, underscoring a robust link between T2DM and ASOM.

## 4 Discussion

Previous studies have identified DM as a potential risk factor for HL (21, 22). However, there are different types of HL, which are often classified by anatomical deficit as conductive, sensorineural, or mixed (1). The disorders of HL are also diverse, such as OE, OM, ASOM, nonsuppurative OM, perforation of the tympanic membrane, hearing difficulty/problems with background noise, sudden idiopathic HL, etc. In this study, to delve deeper into the associations between common hearing disorders and T2DM, we selected 10 common hearing disorders as outcomes. The univariate MR analysis from the NHGRI-EBI and DIAGRAM consortium supports a significant causal association between T2DM and an elevated risk for ASOM. Furthermore, multivariable MR analyses, adjusted for genetically predicted sleep duration, alcohol consumption, body mass index, and smoking, continue to demonstrate a positive causal relationship between T2DM and ASOM. However, a causal link between T2DM and other hearing disorders was either observed only in analyses from a single source or not detected at all. Our study is the first to reveal the relationship between ASOM and T2DM with causal evidence.

It has been reported that in the process of DM and its complications, the body will produce a persistent inflammatory response due to immune cell dysfunction and inflammatory pathway activation (52–54). Patients with DM have hyperglycemia for a long time, which leads to increased plasma osmolarity and inhibition of leukocyte activity (55). In the middle ear (ME) of diabetic patients, inhibition of leukocyte activity leads to a decrease in the internal killing, phagocytosis, and adhesion of leukocytes, which increases the risk of infection. In addition, T2DM is often accompanied by microangiopathy, which is characterized by structural alterations of the capillary walls including thickening of the basement membrane and increased permeability of capillary vessels (56–58). The thickened basement membrane of the capillary wall reduces blood flow to certain areas (59, 60). Capillary damage leads to tissue ischemia and hypoxia, and metabolic disorder. Reducing the normal intake of calories and proteins impairs immune function and reduces antibody production, which in turn inhibits the ability to clear invading pathogens and increases the risk of infection. This may be one of the reasons why T2DM increases the risk of ASOM.

ASOM is a purulent lesion caused by pyogenic bacteria invading the tympanic mucosa through the eustachian tube (ET). ET is a complex structure connecting the middle ear cavity to the nasopharynx (61), and eustachian tube dysfunction (ETD) is considered to be associated with most ME pathologies, although the mechanism of its role in ME diseases is still unclear (62, 63). Recently, the important functions of surfactant protein (SP) in the body have received increasing attention from different specialists. Studies have confirmed the presence of surfactant proteins A and D (SP-A and SP-D) in the human ET (64–66). As the major protein component of surfactant, SP-A plays an important role in innate and acquired immune processes (67). When inflammation occurs, SP-A can promote the recruitment of inflammatory cells, the activation of phagocytic cells, or directly kill pathogens, thereby preventing pathogen infection, regulating allergic reactions, and alleviating inflammation (68–70). This suggests that SP-A may enhance the resistance of ET to infection by participating in immune defense. In adult gerbils and mice, intranasal application of aerosolized metered dose inhaler surfactant reduced the severity and duration of ME infections (71). In addition, SP-A plays a key role in the working process of ET. ETD impairs the clearance of inflammatory products and secretions in the ME and ET by affecting the mucociliary system of ET (62). Recent studies have also shown that SP-D gene polymorphism (rs721917) was associated with gestational DM (72), and the serum SP-D was found to have negative association with extra-pulmonary infections in T2DM patients (73). Therefore, it can be speculated that if the expression of SP in the ET changes, it may lead to the exacerbation of ASOM. This may be another potential mechanism by which T2DM increases the risk of ASOM. However, current studies only shown changes in the expression of SP. The associations between SP, ASOM and ET function have not been investigated. More experimental models are needed to demonstrate the possible mechanisms by which T2DM promotes the development of ASOM by affecting SP.

Our study shows several predominant strengths. Firstly, the causal relationship between T2DM and ASOM was revealed by MR for the first time. MR analysis design can avoid the influence of reverse causation and residual confounding in the process of exploring the causal association between T2DM and ASOM. In addition, the GWAS summary data of T2DM were obtained from two independent European populations, this non-overlapping exposure can avoid the possible bias. Finally, sensitivity analyses with various approaches support the robustness of our MR results. However, there are several certain limitations in our study. The GWAS data of the majority of participants in our study came from European, which makes our results avoid population heterogeneity while also may not be entirely applicable to subjects in other populations. Second, because of the limitations of summarized GWAS data, stratified analyses based on common factors (such as age, sex, hypertension, etc.) could not be performed in our study.

Our findings provide important insights into understanding and preventing diabetes-related complications, particularly ear infections. These findings offer significant insights into understanding and preventing T2DM complications, particularly in the realm of ear infections. They also provide valuable reference information for public health strategies and individual medical decision-making, highlighting the importance and necessity of considering T2DM as a potential risk factor in managing and preventing such complications.

## 5 Conclusion

In summary, our study is the first to reveal the relationship of T2DM and ASOM with causal evidence. The univariate MR analysis from the NHGRI-EBI and DIAGRAM consortium supports a causal association between T2DM and an increased risk of ASOM. MVMR analyses adjusted for confounders continue to demonstrate this association. However, a causal link between T2DM and other hearing disorders was either observed only in analyses from a single source or not detected at all. To further confirm and elucidate the biological mechanisms underpinning the association between T2DM and ASOM, more experimental models are need to perform in future studies.

## Data availability statement

The original contributions presented in the study are included in the article/**Supplementary Material**. Further inquiries can be directed to the corresponding author.

## Author contributions

LK: Conceptualization, Data curation, Methodology, Writing – original draft, Writing – review & editing. CD: Data curation, Formal Analysis, Software, Writing – original draft. JW: Data curation, Software, Validation, Visualization, Writing – original draft, Writing – review & editing. FZ: Formal Analysis, Funding acquisition, Resources, Writing – original draft. BY: Data curation, Formal Analysis, Methodology, Writing – original draft. ZW: Data curation, Formal Analysis, Methodology, Writing – original draft. MY: Data curation, Formal Analysis, Investigation, Methodology, Writing – original draft. CX: Data curation, Formal Analysis, Investigation, Methodology, Writing – original draft. PQ: Conceptualization, Methodology, Supervision, Writing – review & editing, Writing – original draft.

## References

[1] Nieman CL Oh ES. Hearing loss. Ann Intern Med. (2020) 173:ITC81–96. doi: 10.7326/AITC202012010 33253610

[2] Chadha S Kamenov K Cieza A. The world report on hearing, 2021. Bull World Health Organ. (2021) 99:242–A. doi: 10.2471/BLT.21.285643 PMC8085630 33953438

[3] Paul CR Moreno MA. Acute otitis media. JAMA Pediatr. (2020) 174:308. doi: 10.1001/jamapediatrics.2019.5664 31985755

[4] Rijk MH Hullegie S Schilder AGM Kortekaas MF Damoiseaux R Verheij TJM. Incidence and management of acute otitis media in adults: a primary care-based cohort study. Fam Pract. (2021) 38:448–53. doi: 10.1093/fampra/cmaa150 33506857

[5] Shirai N Preciado D. Otitis media: what is new? Curr Opin Otolaryngol Head Neck Surg. (2019) 27:495–8. doi: 10.1097/MOO.0000000000000591 31592792

[6] Leichtle A Hoffmann TK Wigand MC. [Otitis media: definition, pathogenesis, clinical presentation, diagnosis and therapy]. Laryngorhinootologie. (2018) 97:497–508. doi: 10.1055/s-0044-101327 29986368

[7] Morris P. Chronic suppurative otitis media. BMJ Clin Evid. (2012) 2012. PMC3412293 23870746

[8] Monasta L Ronfani L Marchetti F Montico M Vecchi Brumatti L Bavcar A. Burden of disease caused by otitis media: systematic review and global estimates. PloS One. (2012) 7:e36226. doi: 10.1371/journal.pone.0036226 22558393 PMC3340347

[9] Sharma N Jaiswal AA Banerjee PK Garg AK. Complications of chronic suppurative otitis media and their management: A single institution 12 years experience. Indian J Otolaryngol Head Neck Surg. (2015) 67:353–60. doi: 10.1007/s12070-015-0836-5 PMC4678280 26693451

[10] Jacobs AM. Diabetes mellitus. Clin Podiatr Med Surg. (1993) 10:231–48. doi: 10.1016/S0891-8422(23)00601-8 8481881

[11] Cloete L. Diabetes mellitus: an overview of the types, symptoms, complications and management. Nurs Stand. (2022) 37:61–6. doi: 10.7748/ns.2021.e11709 34708622

[12] Magliano DJ Boyko EJ. IDF DIABETES ATLAS. IDF Diabetes Atlas. 10th ed. Brussels: International Diabetes Federation (2021). 35914061

[13] Nanda M Sharma R Mubarik S Aashima A Zhang K. Type-2 diabetes mellitus (T2DM): spatial-temporal patterns of incidence, mortality and attributable risk factors from 1990 to 2019 among 21 world regions. Endocrine. (2022) 77:444–54. doi: 10.1007/s12020-022-03125-5 35841511

[14] Seiglie JA Marcus ME Ebert C Prodromidis N Geldsetzer P Theilmann M. Diabetes prevalence and its relationship with education, wealth, and BMI in 29 low- and middle-income countries. Diabetes Care. (2020) 43:767–75. doi: 10.2337/dc19-1782 PMC7085810 32051243

[15] Fletcher B Gulanick M Lamendola C. Risk factors for type 2 diabetes mellitus. J Cardiovasc Nurs. (2002) 16:17–23. doi: 10.1097/00005082-200201000-00003 11800065

[16] Kolb H Martin S. Environmental/lifestyle factors in the pathogenesis and prevention of type 2 diabetes. BMC Med. (2017) 15:131. doi: 10.1186/s12916-017-0901-x 28720102 PMC5516328

[17] Zheng Y Ley SH Hu FB. Global aetiology and epidemiology of type 2 diabetes mellitus and its complications. Nat Rev Endocrinol. (2018) 14:88–98. doi: 10.1038/nrendo.2017.151 29219149

[18] Damanik J Yunir E. Type 2 diabetes mellitus and cognitive impairment. Acta Med Indones. (2021) 53:213–20. 34251351

[19] Taylor R. Type 2 diabetes: etiology and reversibility. Diabetes Care. (2013) 36:1047–55. doi: 10.2337/dc12-1805 PMC3609491 23520370

[20] Vicente-Herrero MT Ramirez-Iniguez de la Torre MV Delgado-Bueno S. Diabetes mellitus and work. Assessment and questionnaires revision. Endocrinol Diabetes Nutr (Engl Ed). (2019) 66:520–7. doi: 10.1016/j.endien.2019.02.007 30979608

[21] Deng Y Chen S Hu J. Diabetes mellitus and hearing loss. Mol Med. (2023) 29:141. doi: 10.1186/s10020-023-00737-z 37875793 PMC10599066

[22] Horikawa C Kodama S Tanaka S Fujihara K Hirasawa R Yachi Y. Diabetes and risk of hearing impairment in adults: a meta-analysis. J Clin Endocrinol Metab. (2013) 98:51–8. doi: 10.1210/jc.2012-2119 23150692

[23] Samocha-Bonet D Wu B Ryugo DK. Diabetes mellitus and hearing loss: A review. Ageing Res Rev. (2021) 71:101423. doi: 10.1016/j.arr.2021.101423 34384902

[24] Ren J Ma F Zhou Y Xu A Zhang J Ma R. Hearing impairment in type 2 diabetics and patients with early diabetic nephropathy. J Diabetes Complications. (2018) 32:575–9. doi: 10.1016/j.jdiacomp.2018.03.014 29776866

[25] Smith TL Raynor E Prazma J Buenting JE Pillsbury HC. Insulin-dependent diabetic microangiopathy in the inner ear. Laryngoscope. (1995) 105:236–40. doi: 10.1288/00005537-199503000-00002 7877409

[26] Burgess S Small DS Thompson SG. A review of instrumental variable estimators for Mendelian randomization. Stat Methods Med Res. (2017) 26:2333–55. doi: 10.1177/0962280215597579 PMC5642006 26282889

[27] Sakaue S Kanai M Tanigawa Y Karjalainen J Kurki M Koshiba S. A cross-population atlas of genetic associations for 220 human phenotypes. Nat Genet. (2021) 53:1415–24. doi: 10.1038/s41588-021-00931-x PMC12208603 34594039

[28] Morris AP Voight BF Teslovich TM Ferreira T Segrè AV Steinthorsdottir V. Large-scale association analysis provides insights into the genetic architecture and pathophysiology of type 2 diabetes. Nat Genet. (2012) 44:981–90. doi: 10.1038/ng.2383 PMC3442244 22885922

[29] Vaucher J Keating BJ Lasserre AM Gan W Lyall DM Ward J. Cannabis use and risk of schizophrenia: a Mendelian randomization study. Mol Psychiatry. (2018) 23:1287–92. doi: 10.1038/mp.2016.252 PMC5984096 28115737

[30] Buxton OM Marcelli E. Short and long sleep are positively associated with obesity, diabetes, hypertension, and cardiovascular disease among adults in the United States. Soc Sci Med. (2010) 71:1027–36. doi: 10.1016/j.socscimed.2010.05.041 20621406

[31] Dawes P Cruickshanks KJ Moore DR Edmondson-Jones M McCormack A Fortnum H. Cigarette smoking, passive smoking, alcohol consumption, and hearing loss. J Assoc Res Otolaryngol. (2014) 15:663–74. doi: 10.1007/s10162-014-0461-0 PMC4141428 24899378

[32] Froehlich P Collet L Valatx JL Morgon A. Sleep and active cochlear micromechanical properties in human subjects. Hear Res. (1993) 66:1–7. doi: 10.1016/0378-5955(93)90254-X 8473241

[33] Hu H Tomita K Kuwahara K Yamamoto M Uehara A Kochi T. Obesity and risk of hearing loss: A prospective cohort study. Clin Nutr. (2020) 39:870–5. doi: 10.1016/j.clnu.2019.03.020 30954364

[34] Ikuta T Stansberry TE Lowe RO. Sleep duration is associated with auditory radiation microstructure. Neurol Res. (2020) 42:739–43. doi: 10.1080/01616412.2020.1773603 32544374

[35] Ng R Sutradhar R Yao Z Wodchis WP Rosella LC. Smoking, drinking, diet and physical activity-modifiable lifestyle risk factors and their associations with age to first chronic disease. Int J Epidemiol. (2020) 49:113–30. doi: 10.1093/ije/dyz078 PMC7124486 31329872

[36] Selman A Burns S Reddy AP Culberson J Reddy PH. The role of obesity and diabetes in dementia. Int J Mol Sci. (2022) 23(16):9267. doi: 10.3390/ijms23169267 36012526 PMC9408882

[37] Lawlor DA Harbord RM Sterne JA Timpson N Davey Smith G. Mendelian randomization: using genes as instruments for making causal inferences in epidemiology. Stat Med. (2008) 27:1133–63. doi: 10.1002/sim.3034 17886233

[38] Pierce BL Ahsan H Vanderweele TJ. Power and instrument strength requirements for Mendelian randomization studies using multiple genetic variants. Int J Epidemiol. (2011) 40:740–52. doi: 10.1093/ije/dyq151 PMC3147064 20813862

[39] Kurki MI Karjalainen J Palta P Sipilä TP Kristiansson K Donner KM. FinnGen provides genetic insights from a well-phenotyped isolated population. Nature. (2023) 613:508–18. doi: 10.1038/s41586-022-05473-8 PMC9849126 36653562

[40] Jones SE Tyrrell J Wood AR Beaumont RN Ruth KS Tuke MA. Genome-wide association analyses in 128,266 individuals identifies new morningness and sleep duration loci. PloS Genet. (2016) 12:e1006125. doi: 10.1371/journal.pgen.1006125 27494321 PMC4975467

[41] Lyon M Andrews SJ Elsworth B Gaunt TR Hemani G Marcora E. The variant call format provides efficient and robust storage of GWAS summary statistics. Genome Biol. (2021) 22(1):32. doi: 10.1186/s13059-020-02248-0 33441155 PMC7805039

[42] Hoffmann TJ Choquet H Yin J Banda Y Kvale MN Glymour M. A large multiethnic genome-wide association study of adult body mass index identifies novel loci. Genetics. (2018) 210:499–515. doi: 10.1534/genetics.118.301479 30108127 PMC6216593

[43] Loh PR Kichaev G Gazal S Schoech AP Price AL. Mixed-model association for biobank-scale datasets. Nat Genet. (2018) 50:906–8. doi: 10.1038/s41588-018-0144-6 PMC6309610 29892013

[44] Burgess S Butterworth A Thompson SG. Mendelian randomization analysis with multiple genetic variants using summarized data. Genet Epidemiol. (2013) 37:658–65. doi: 10.1002/gepi.21758 PMC4377079 24114802

[45] Greco MF Minelli C Sheehan NA Thompson JR. Detecting pleiotropy in Mendelian randomisation studies with summary data and a continuous outcome. Stat Med. (2015) 34:2926–40. doi: 10.1002/sim.6522 25950993

[46] Bowden J Davey Smith G Burgess S. Mendelian randomization with invalid instruments: effect estimation and bias detection through Egger regression. Int J Epidemiol. (2015) 44:512–25. doi: 10.1093/ije/dyv080 PMC4469799 26050253

[47] Bowden J Davey Smith G Haycock PC Burgess S. Consistent estimation in mendelian randomization with some invalid instruments using a weighted median estimator. Genet Epidemiol. (2016) 40:304–14. doi: 10.1002/gepi.21965 PMC4849733 27061298

[48] Verbanck M Chen CY Neale B Do R. Detection of widespread horizontal pleiotropy in causal relationships inferred from Mendelian randomization between complex traits and diseases. Nat Genet. (2018) 50:693–8. doi: 10.1038/s41588-018-0099-7 PMC6083837 29686387

[49] Burgess S Thompson SG. Mendelian randomization. 2nd Edition. New York: Chapman and Hall/CRC (2021). doi: 10.1201/9780429324352

[50] Liang Y Wu D Ledesma D Davis C Slaughter R Guo Z. Virtual Tai-Chi System: A smart-connected modality for rehabilitation. Smart Health. (2018) 9-10:232–49. doi: 10.1016/j.smhl.2018.07.021

[51] Amidei C. Symptom-based interventions to promote quality survivorship. Neuro-Oncology. (2018) 20:VII27–39. doi: 10.1093/neuonc/noy100 PMC6225746 29905840

[52] Boutens L Hooiveld GJ Dhingra S Cramer RA Netea MG Stienstra R. Unique metabolic activation of adipose tissue macrophages in obesity promotes inflammatory responses. Diabetologia. (2018) 61:942–53. doi: 10.1007/s00125-017-4526-6 PMC6448980 29333574

[53] Klopf J Brostjan C Eilenberg W Neumayer C. Neutrophil extracellular traps and their implications in cardiovascular and inflammatory disease. Int J Mol Sci. (2021) 22(2):559. doi: 10.3390/ijms22020559 33429925 PMC7828090

[54] Lontchi-Yimagou E Sobngwi E Matsha TE Kengne AP. Diabetes mellitus and inflammation. Curr Diabetes Rep. (2013) 13:435–44. doi: 10.1007/s11892-013-0375-y 23494755

[55] Zhang H Yang Z Zhang W Niu Y Li X Qin L. White blood cell subtypes and risk of type 2 diabetes. J Diabetes Complications. (2017) 31:31–7. doi: 10.1016/j.jdiacomp.2016.10.029 27863973

[56] Avogaro A Albiero M Menegazzo L de Kreutzenberg S Fadini GP. Endothelial dysfunction in diabetes: the role of reparatory mechanisms. Diabetes Care. (2011) 34 Suppl 2:S285–90. doi: 10.2337/dc11-s239 PMC3632194 21525470

[57] Jandl K Marsh LM Hoffmann J Mutgan AC Baum O Bloch W. Basement membrane remodeling controls endothelial function in idiopathic pulmonary arterial hypertension. Am J Respir Cell Mol Biol. (2020) 63:104–17. doi: 10.1165/rcmb.2019-0303OC 32160015

[58] Palladino R Tabak AG Khunti K Valabhji J Majeed A Millett C. Association between pre-diabetes and microvascular and macrovascular disease in newly diagnosed type 2 diabetes. BMJ Open Diabetes Res Care. (2020) 8(1):e001061. doi: 10.1136/bmjdrc-2019-001061 PMC7202749 32332069

[59] Genes SG Genes VS. [Diabetic neuropathy and lesion of the vessels]. Arkh Patol. (1981) 43:77–82. 7271501

[60] Nukada H. Ischemia and diabetic neuropathy. Handb Clin Neurol. (2014) 126:469–87. doi: 10.1016/B978-0-444-53480-4.00023-0 25410240

[61] Tysome JR Sudhoff H. The role of the eustachian tube in middle ear disease. Adv Otorhinolaryngol. (2018) 81:146–52. doi: 10.1159/000485581 29794454

[62] Maddineni S Ahmad I. Updates in eustachian tube dysfunction. Otolaryngol Clin North Am. (2022) 55:1151–64. doi: 10.1016/j.otc.2022.07.010 36371132

[63] Schilder AG Bhutta MF Butler CC Holy C Levine LH Kvaerner KJ. Eustachian tube dysfunction: consensus statement on definition, types, clinical presentation and diagnosis. Clin Otolaryngol. (2015) 40:407–11. doi: 10.1111/coa.12475 PMC4600223 26347263

[64] Kankavi O. Immunodetection of surfactant proteins in human organ of Corti, Eustachian tube and kidney. Acta Biochim Pol. (2003) 50:1057–64. doi: 10.18388/abp.2003_3631 14739994

[65] Paananen R Glumoff V Hallman M. Surfactant protein A and D expression in the porcine Eustachian tube. FEBS Lett. (1999) 452:141–4. doi: 10.1016/S0014-5793(99)00602-X 10386578

[66] Paananen R Sormunen R Glumoff V van Eijk M Hallman M. Surfactant proteins A and D in Eustachian tube epithelium. Am J Physiol Lung Cell Mol Physiol. (2001) 281:L660–7. doi: 10.1152/ajplung.2001.281.3.L660 11504694

[67] Watson A Madsen J Clark HW. SP-A and SP-D: dual functioning immune molecules with antiviral and immunomodulatory properties. Front Immunol. (2020) 11:622598. doi: 10.3389/fimmu.2020.622598 33542724 PMC7851053

[68] Abdel-Razek O Ni L Yang F Wang G. Innate immunity of surfactant protein A in experimental otitis media. Innate Immun. (2019) 25:391–400. doi: 10.1177/1753425919866006 31378117 PMC6900641

[69] Chen T Li G Liu W Fan Z Li L. Surfactant protein A can affect macrophage phagocytosis: an important pathogenic mechanism of otitis media with effusion. J Assoc Res Otolaryngol. (2023) 24:171–80. doi: 10.1007/s10162-023-00893-3 PMC10121950 36820988

[70] Francisco D Wang Y Conway M Hurbon AN Dy ABC Addison KJ. Surfactant protein-A protects against IL-13-induced inflammation in asthma. J Immunol. (2020) 204:2829–39. doi: 10.4049/jimmunol.1901227 PMC7304346 32245819

[71] Chandrasekhar SS Mautone AJ. Otitis media: treatment with intranasal aerosolized surfactant. Laryngoscope. (2004) 114:472–85. doi: 10.1097/00005537-200403000-00017 15091221

[72] Xu J Chen Y Tang L Teng X Feng L Jin L. Association of surfactant protein D gene polymorphism with susceptibility to gestational diabetes mellitus: a case-control study. BMC Pregnancy Childbirth. (2022) 22:231. doi: 10.1186/s12884-022-04541-1 35317741 PMC8939171

[73] Jawed S Atta K Zia S Altaf B. Relationship of surfactant protein-D with random blood glucose and extra pulmonary infections in type-2 diabetes mellitus. J Pak Med Assoc. (2021) 71:195–200. doi: 10.47391/JPMA.818 35157648

