# Supplementary material for: Causal association between type 2 diabetes mellitus and acute suppurative otitis media: insights from a univariate and multivariate Mendelian randomization study
Source: Front Endocrinol (Lausanne). 2024 May 21;15:1407503. doi: 10.3389/fendo.2024.1407503 (PMC11148255; doi:10.3389/fendo.2024.1407503)
Supplement: Supplementary file 1 [file DataSheet_1.docx]

Supplementary Figures

## Figure S1. Leave-one-out sensitivity analysis for T2DM derived from NHGRI-EBI under genome-wide significance threshold instrumental variables.

(A) T2DM on conductive hearing loss;

(B) T2DM on otitis externa;

(C) T2DM on acute suppurative otitis media;

(D) T2DM on nonsuppurative otitis media;

(E) T2DM on sensorineural hearing loss;

(F) T2DM on perforation of tympanic membrane;

(G) T2DM on hearing difficulty/problems with background noise;

(H) T2DM on sudden idiopathic hearing loss;

(I) T2DM on mixed conductive and sensorineural hearing loss;

(J) T2DM on otitis media.


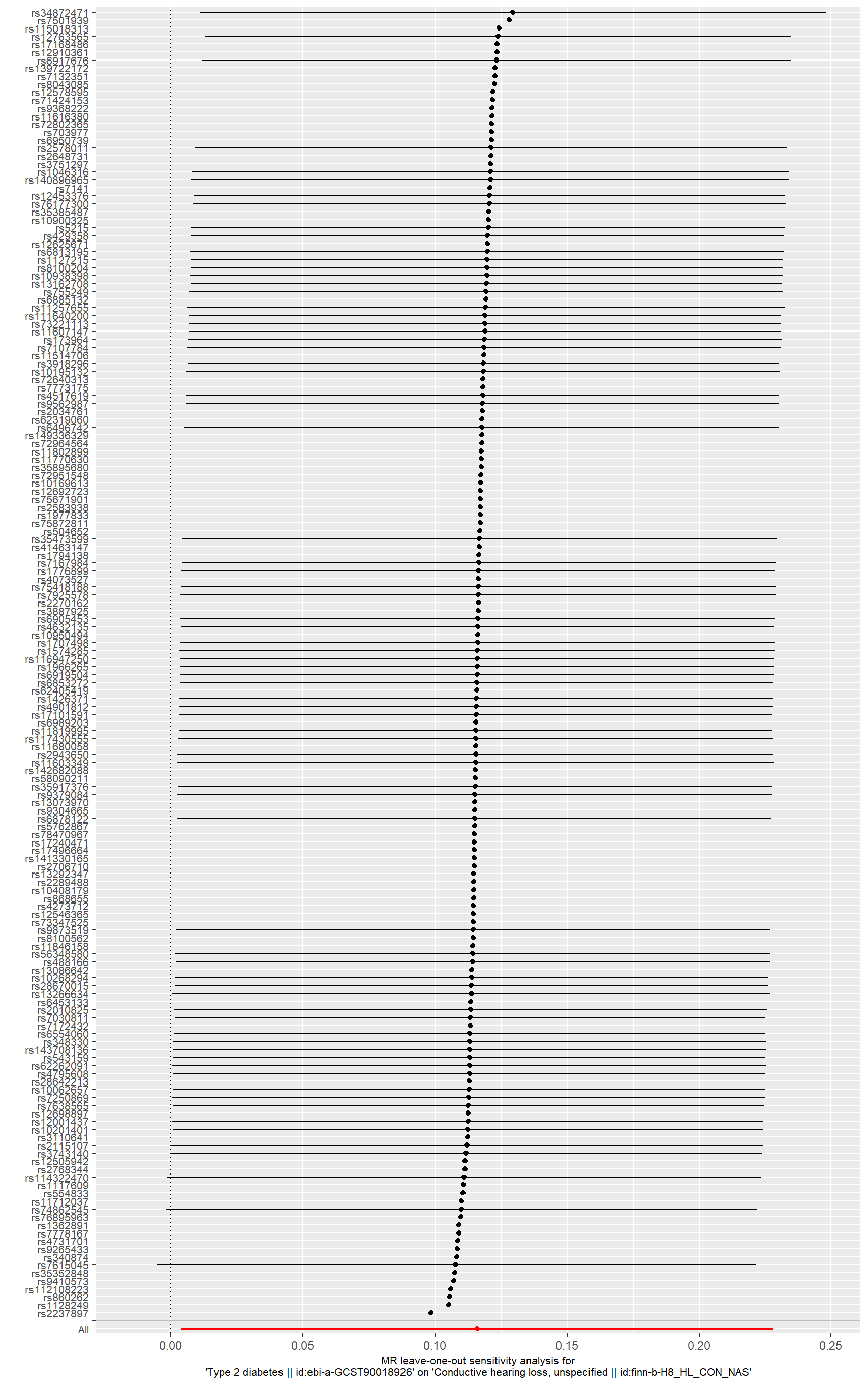


(A)


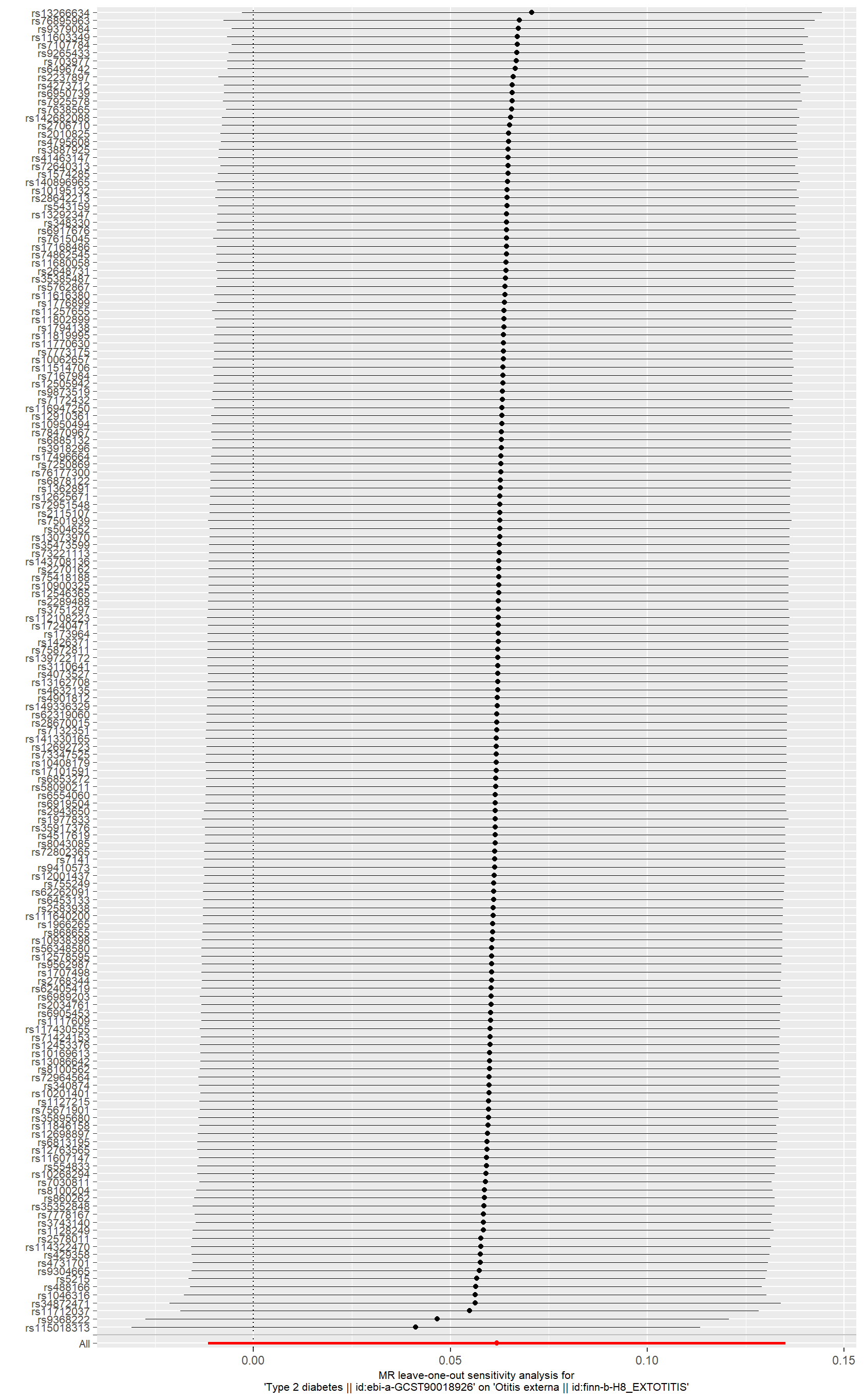


(B)


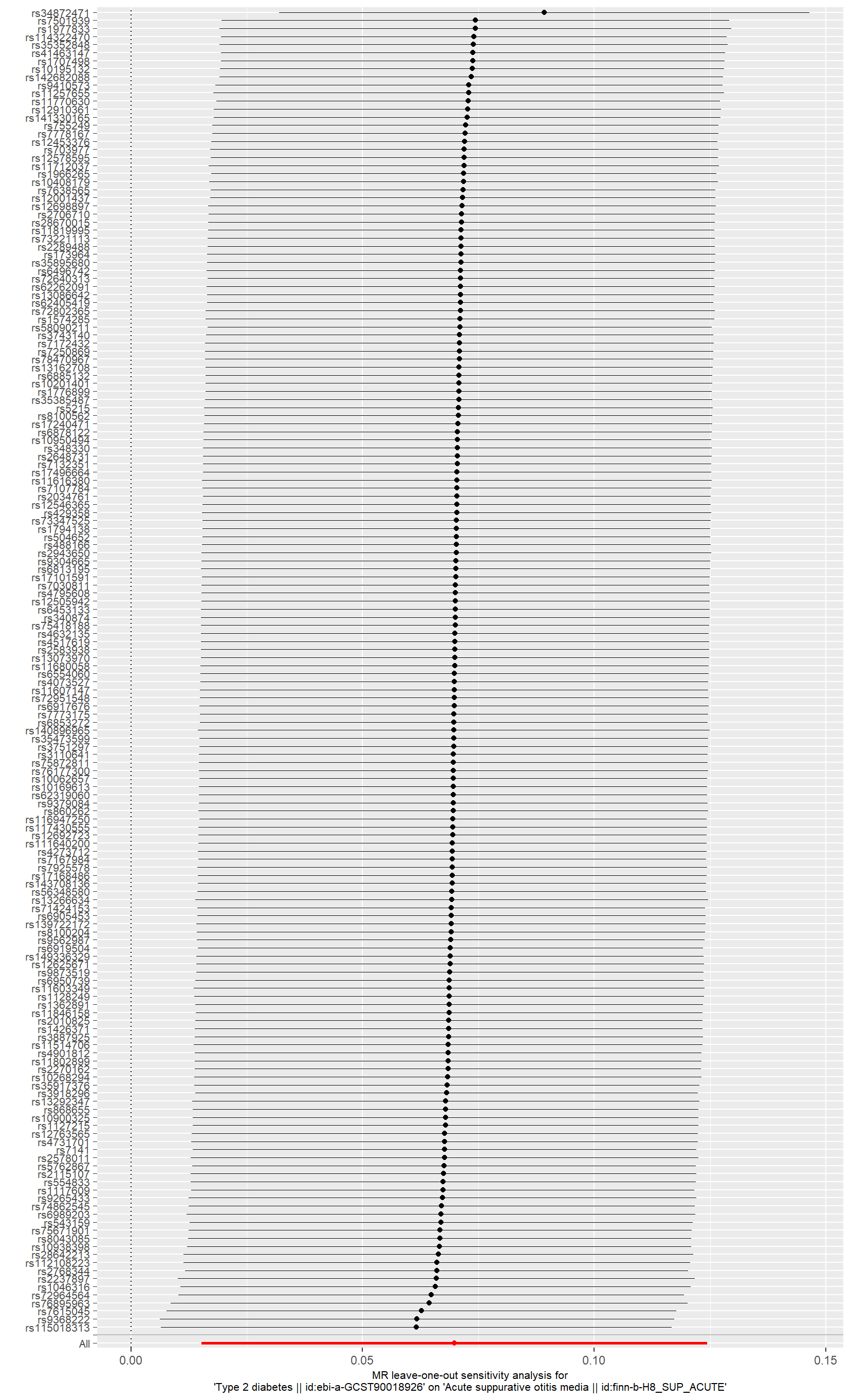


(C)


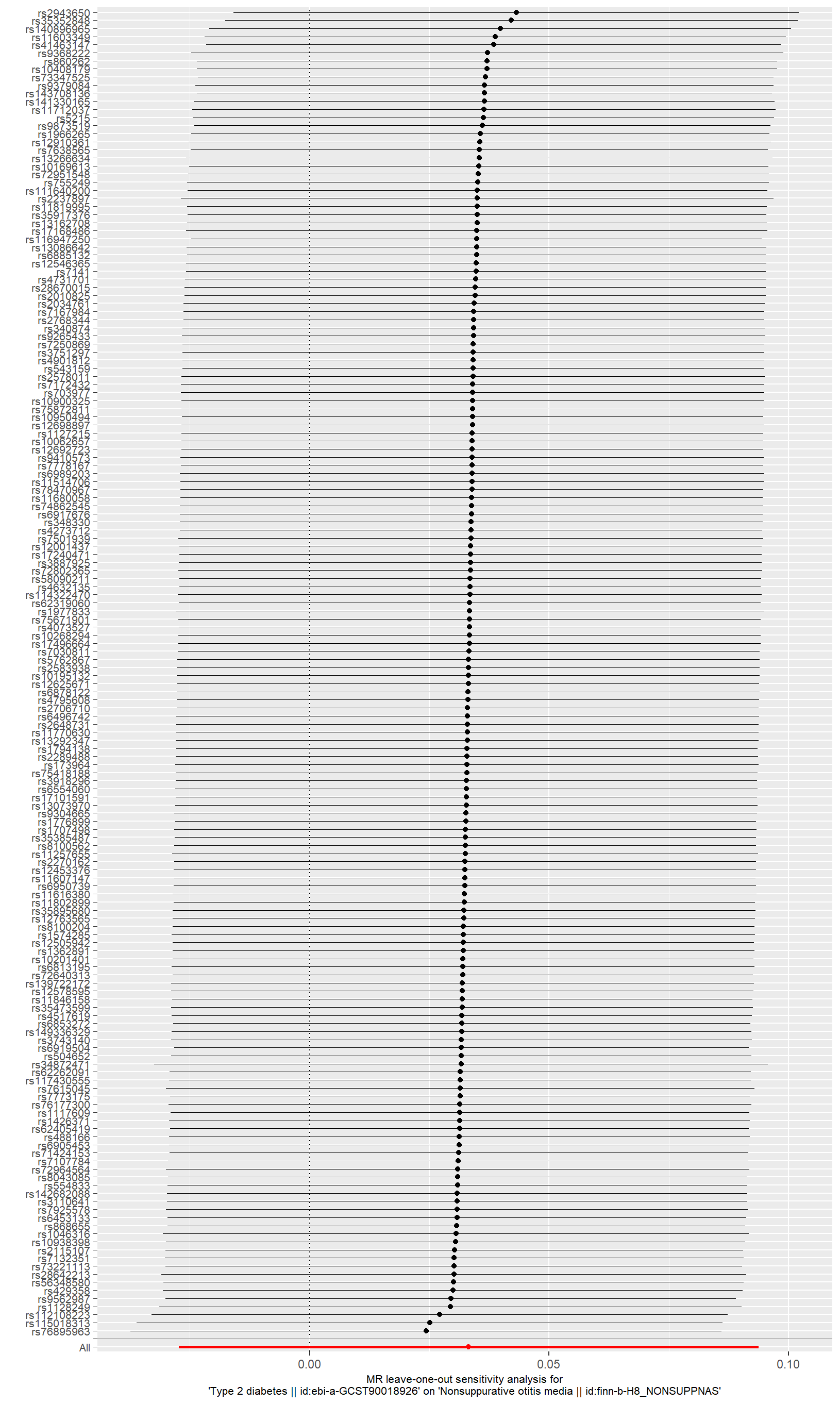


(D)


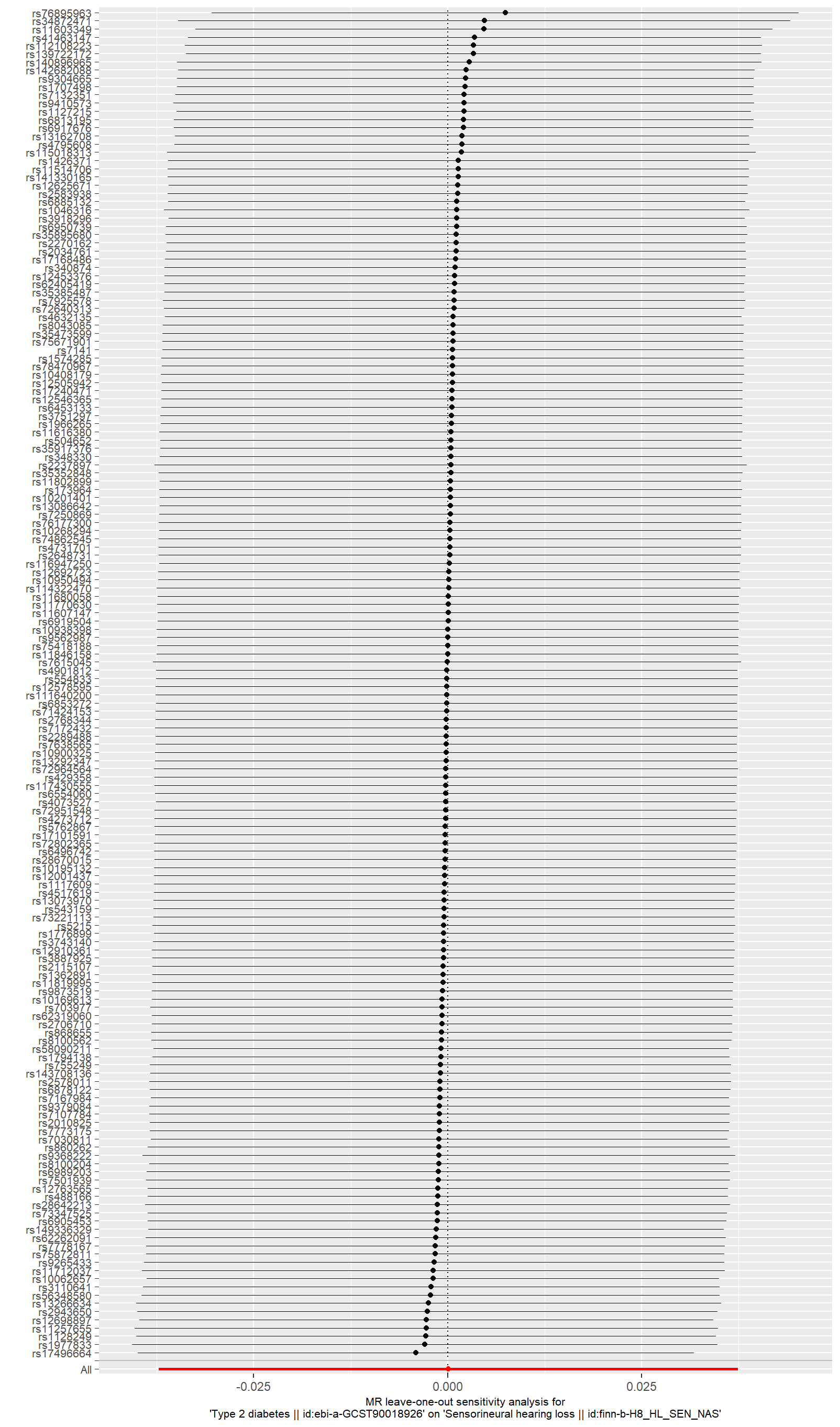


(E)


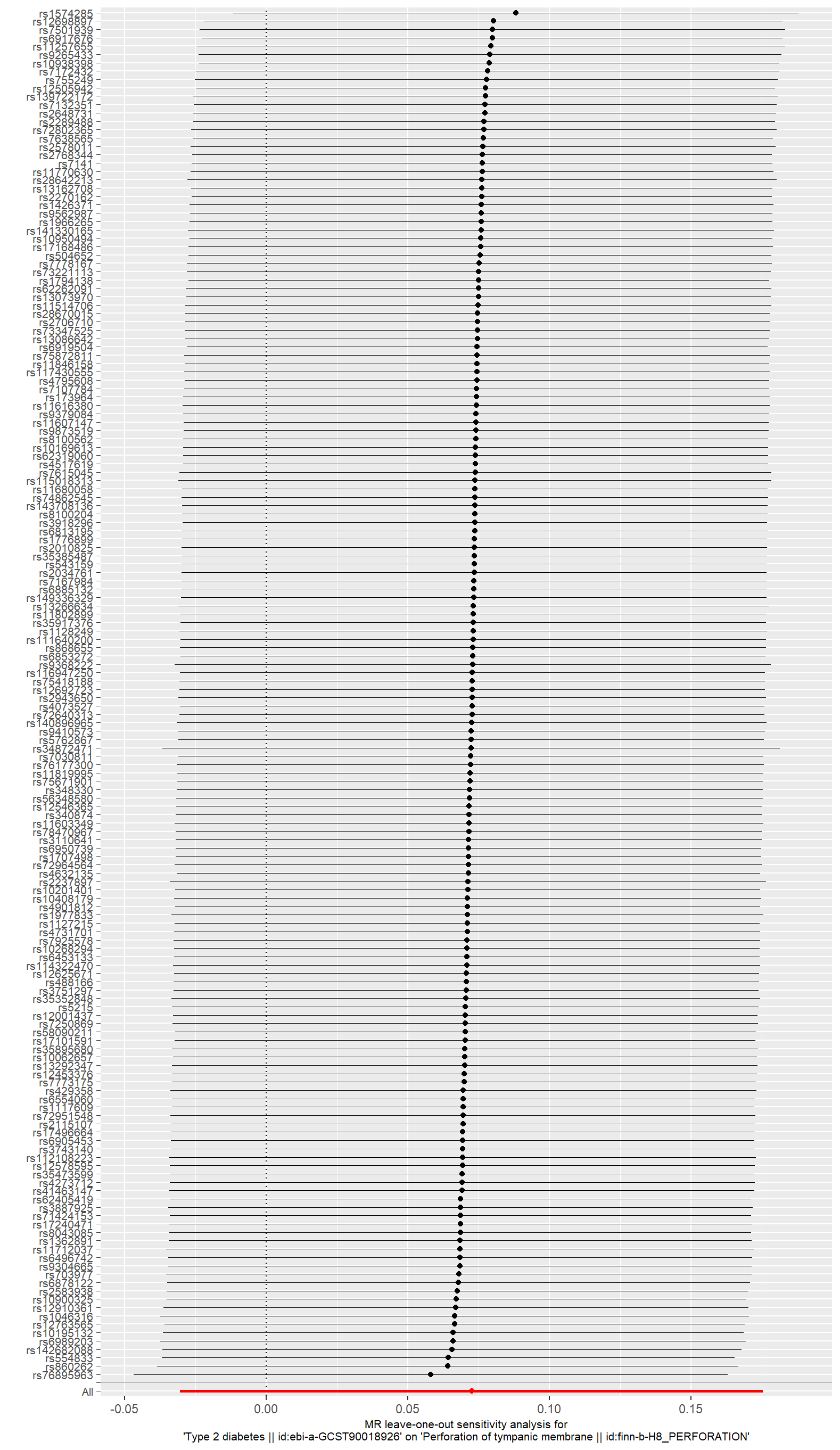


(F)


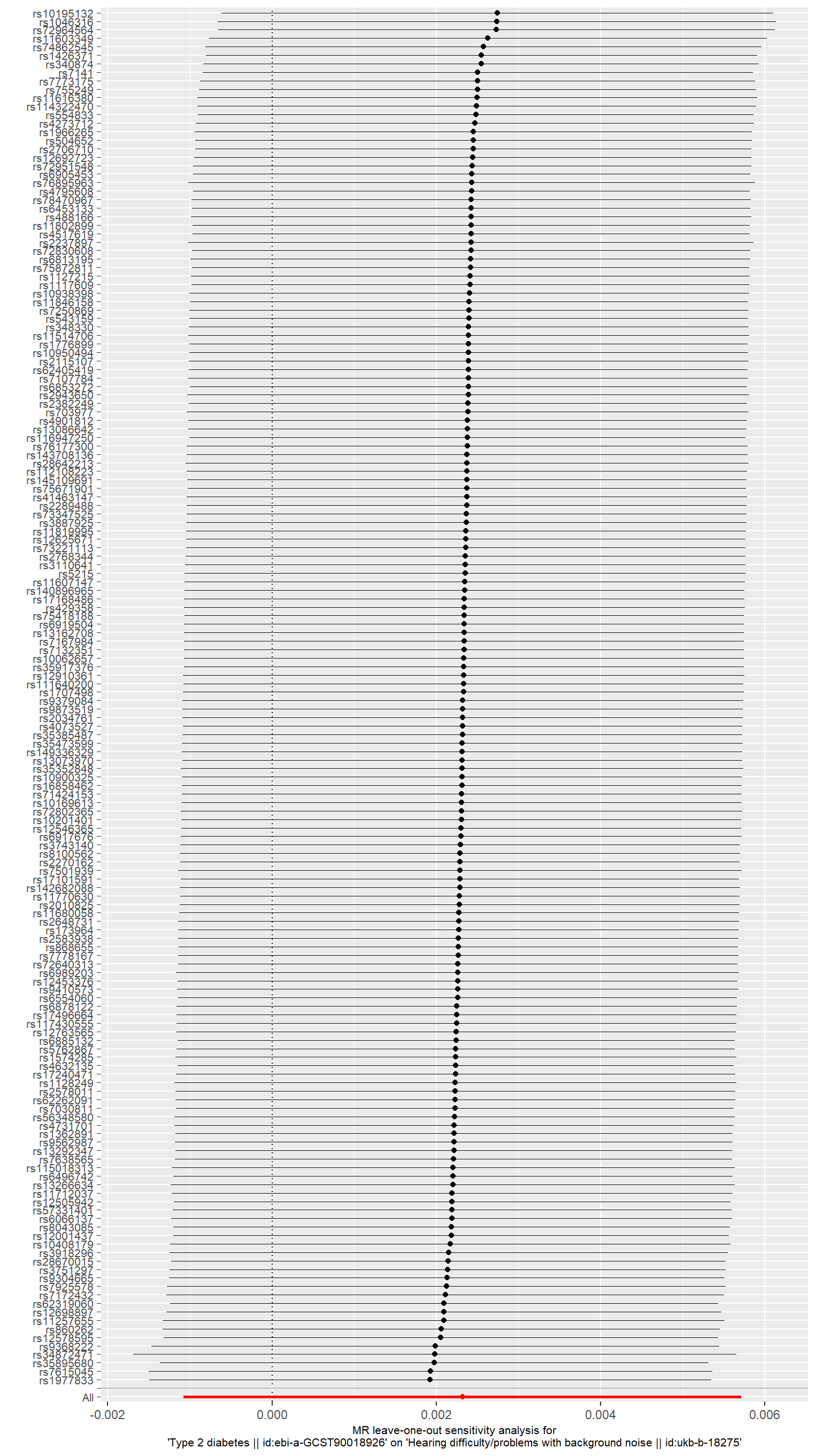


(G)


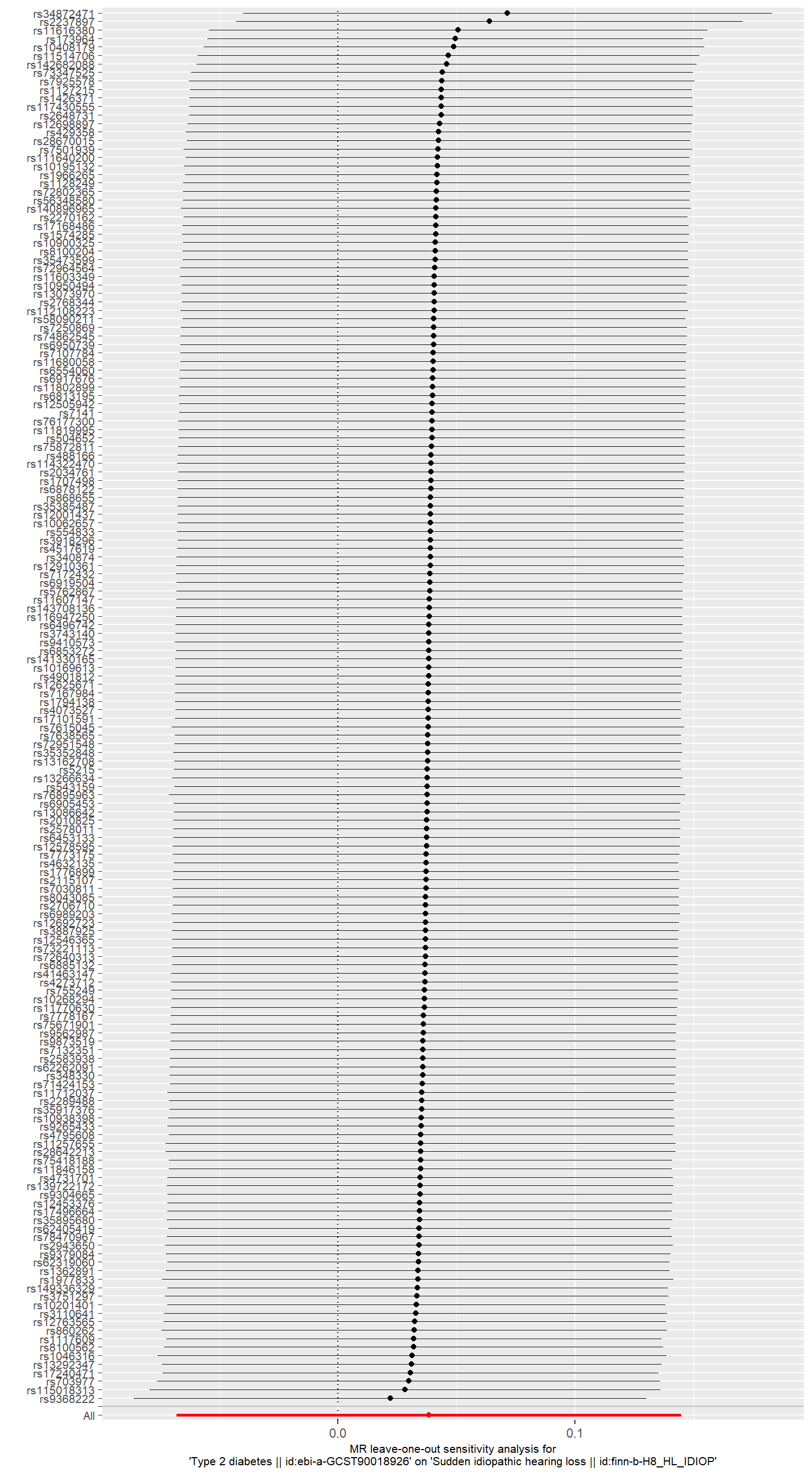


(H)


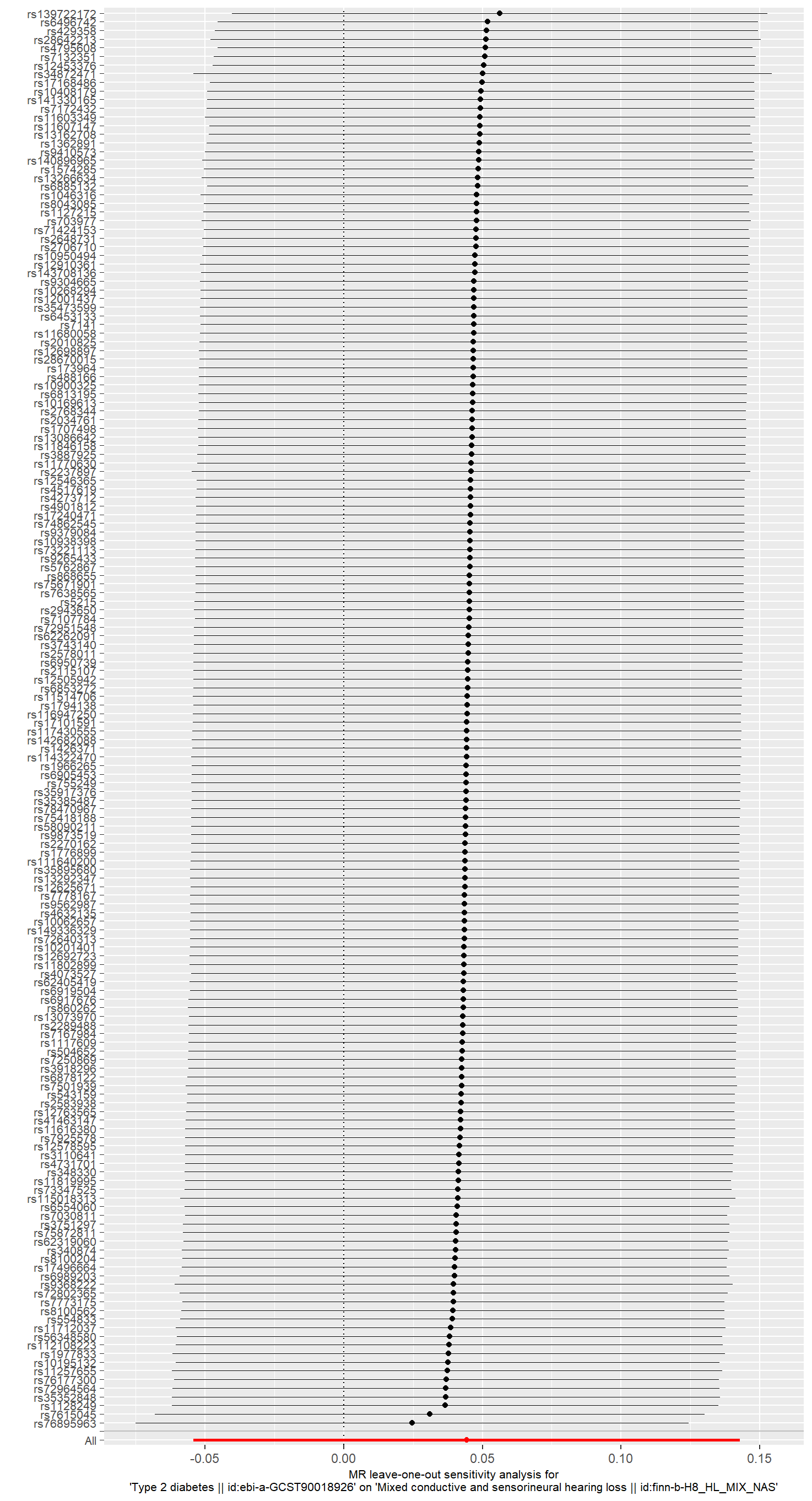


(I)


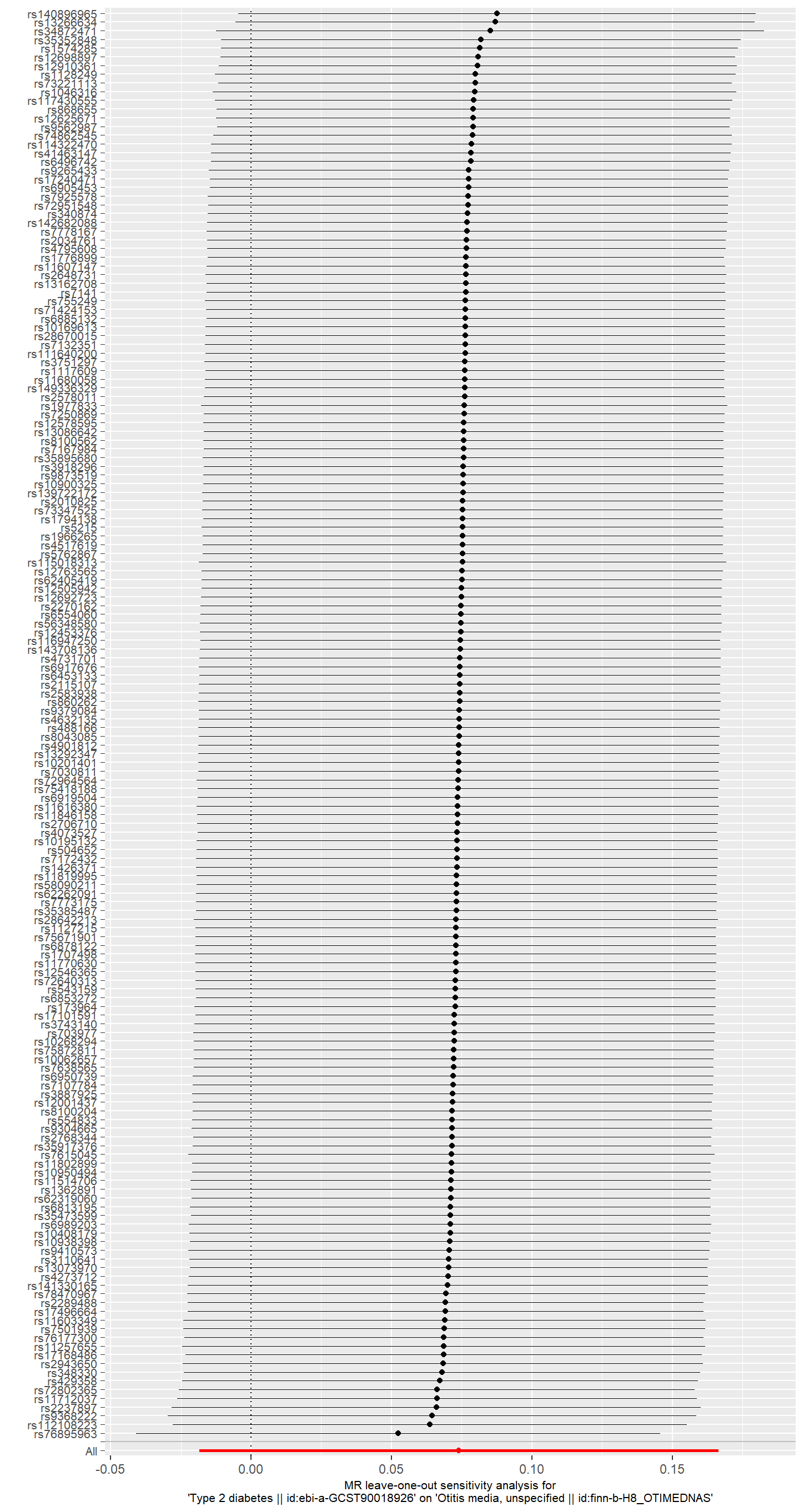


(J)

## Figure S2. Leave-one-out sensitivity analysis for T2DM derived from DIAGRAM under genome-wide significance threshold instrumental variables.

(A) T2DM on conductive hearing loss;

(B) T2DM on otitis externa;

(C) T2DM on acute suppurative otitis media;

(D) T2DM on nonsuppurative otitis media;

(E) T2DM on sensorineural hearing loss;

(F) T2DM on perforation of tympanic membrane;

(G) T2DM on hearing difficulty/problems with background noise;

(H) T2DM on sudden idiopathic hearing loss;

(I) T2DM on mixed conductive and sensorineural hearing loss;

(J) T2DM on otitis media.


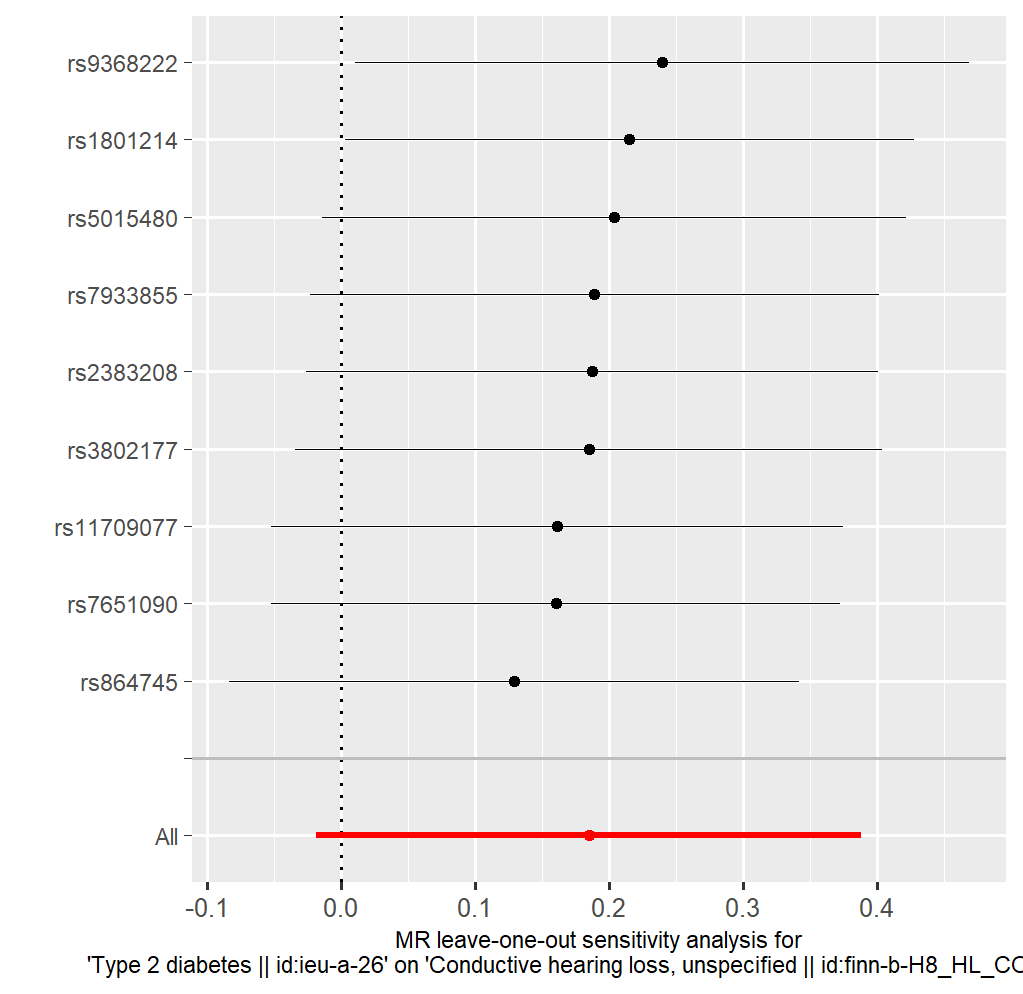


(A)


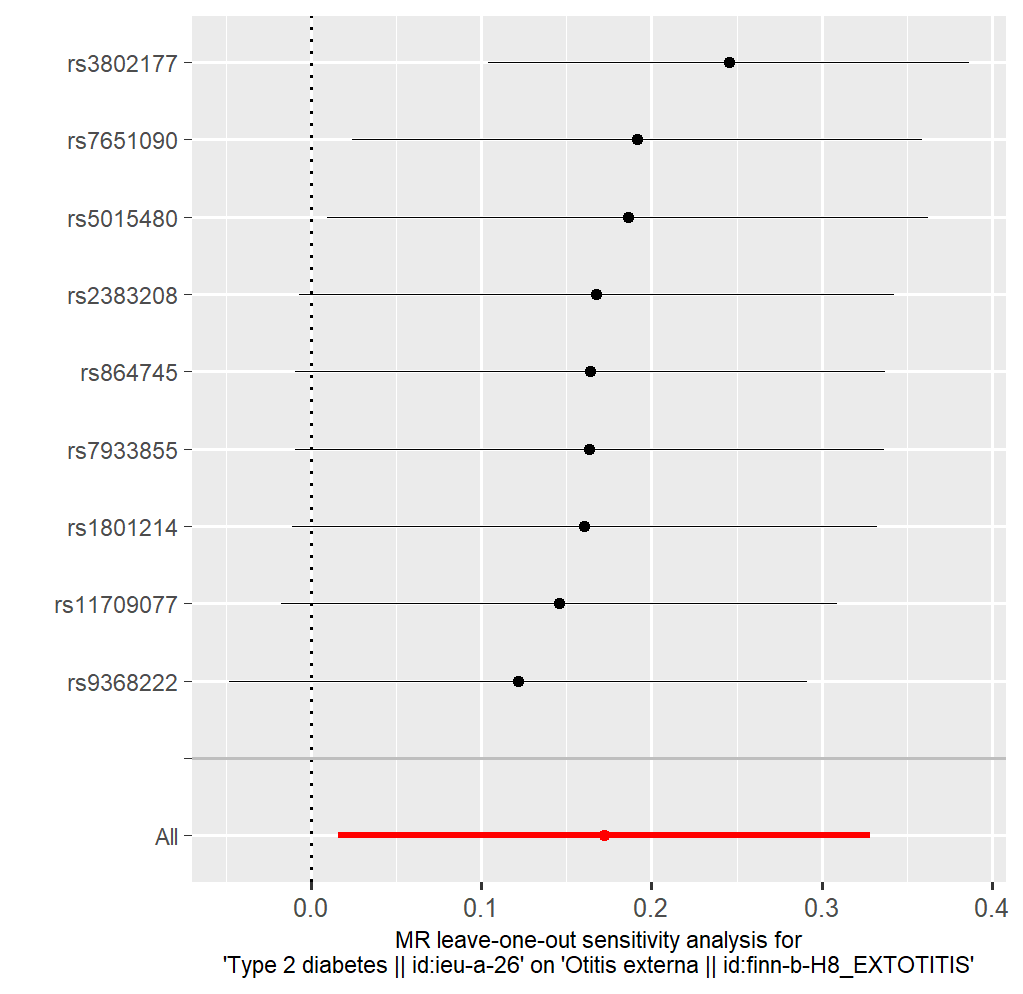


(B)


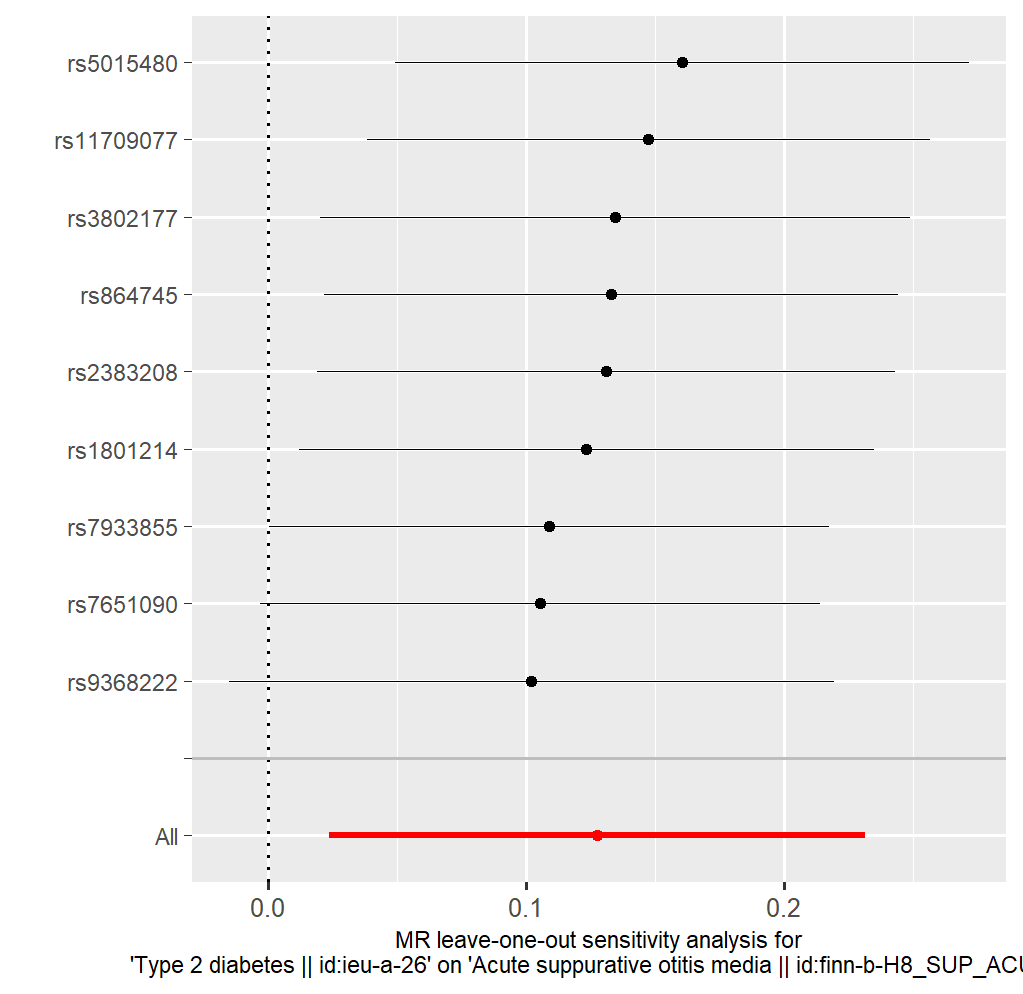


(C)


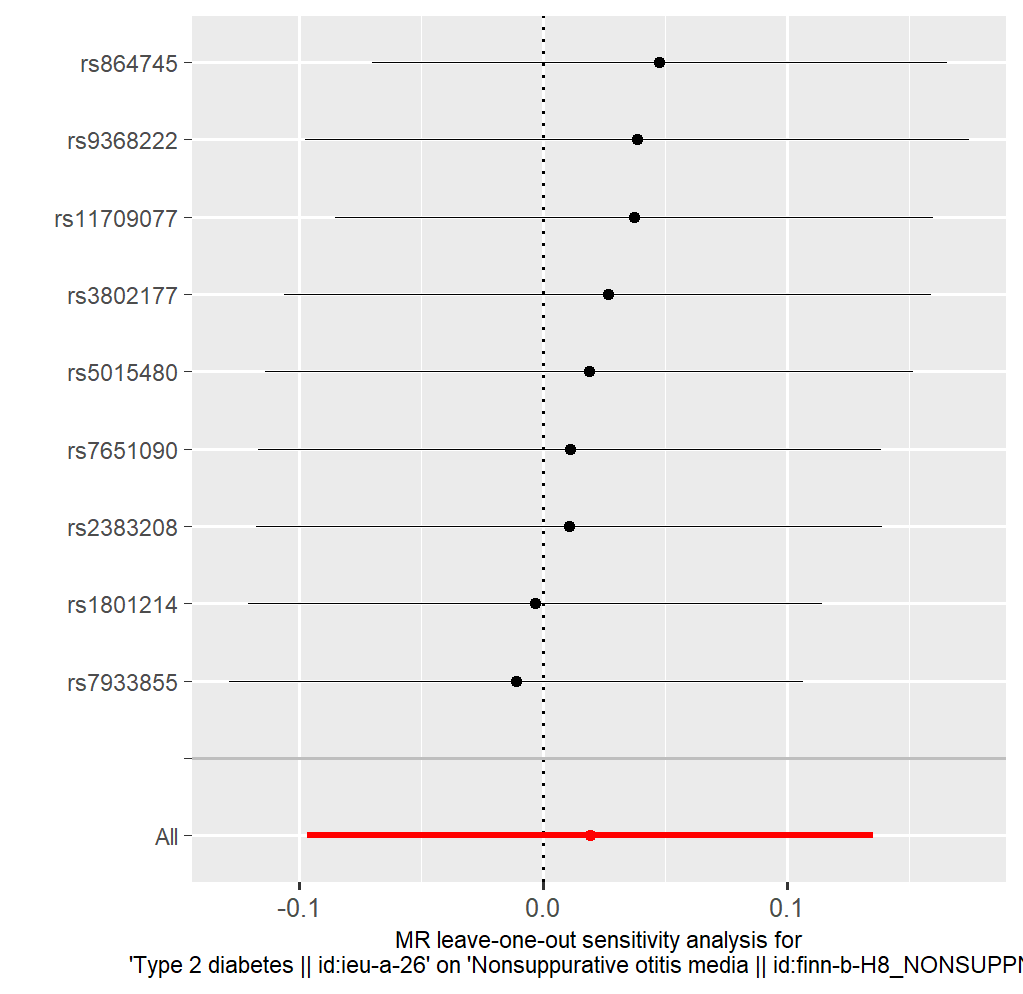


(D)


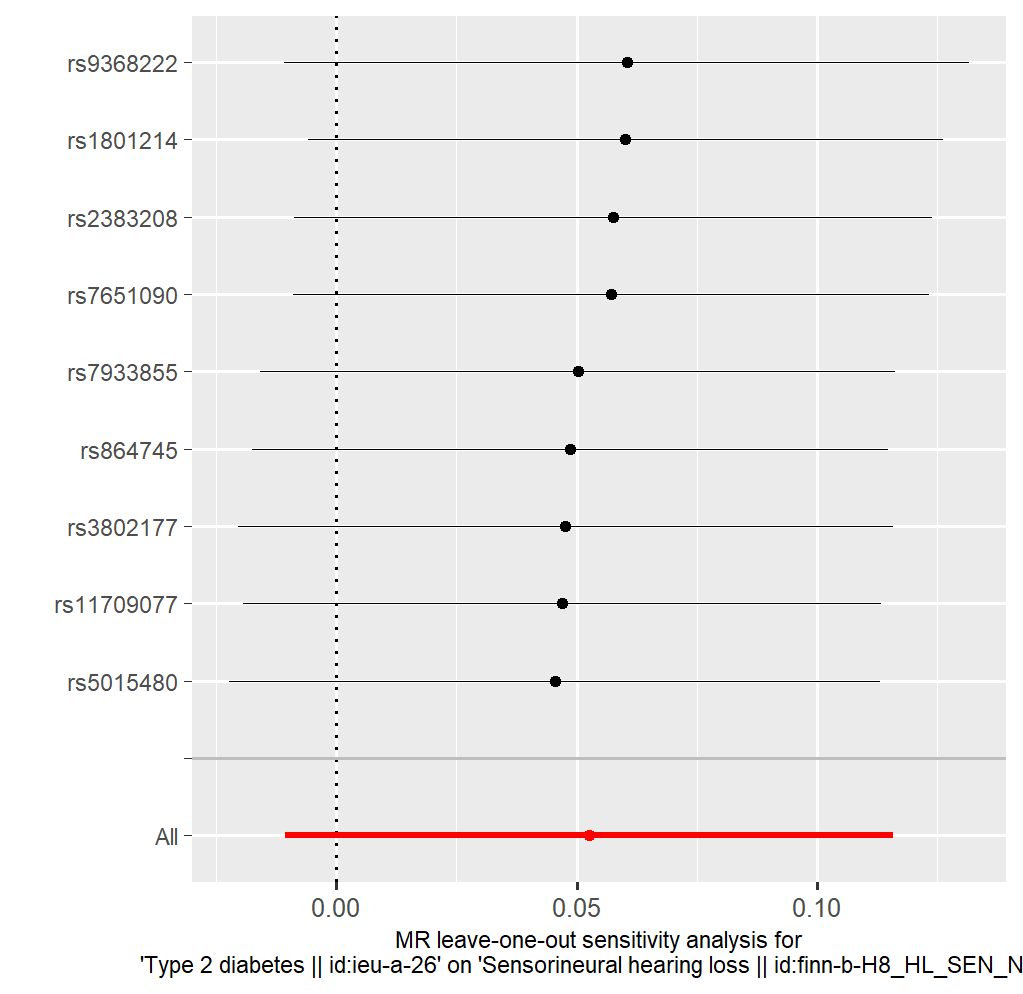


(E)


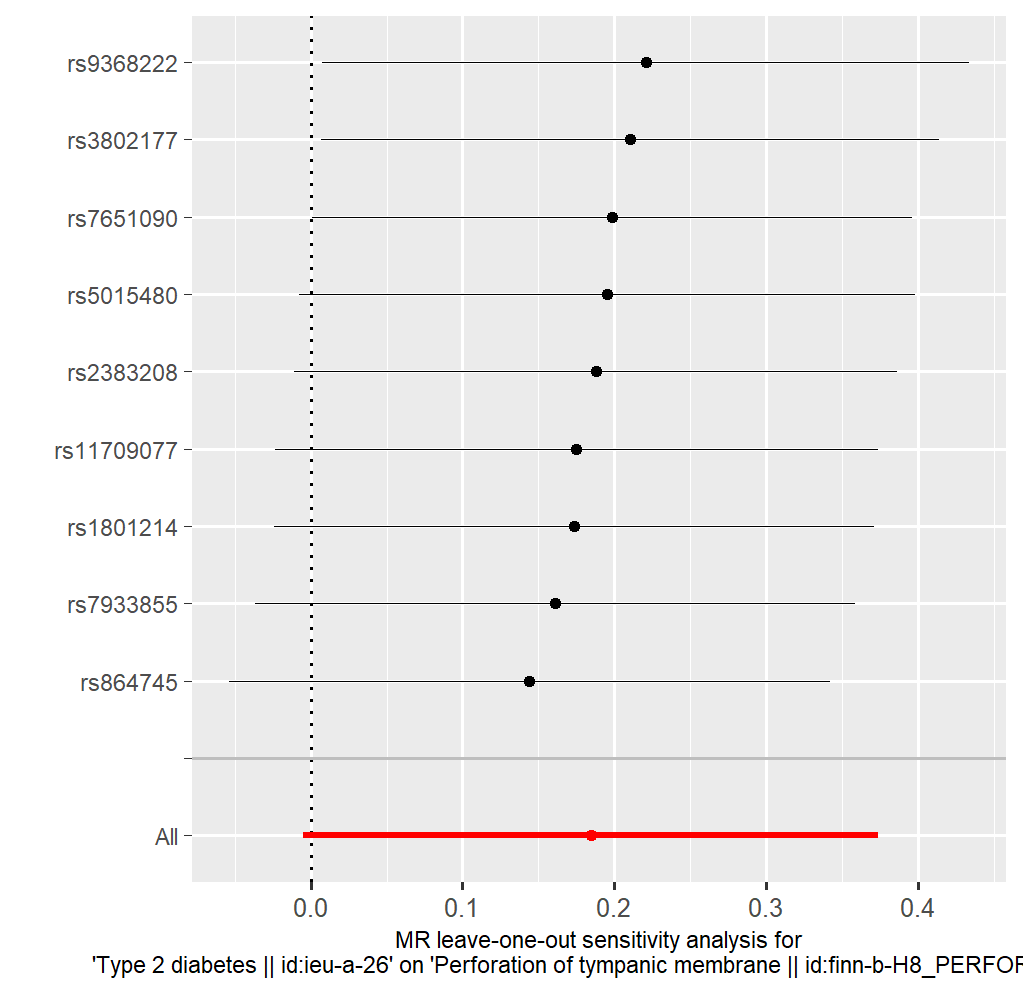


(F)


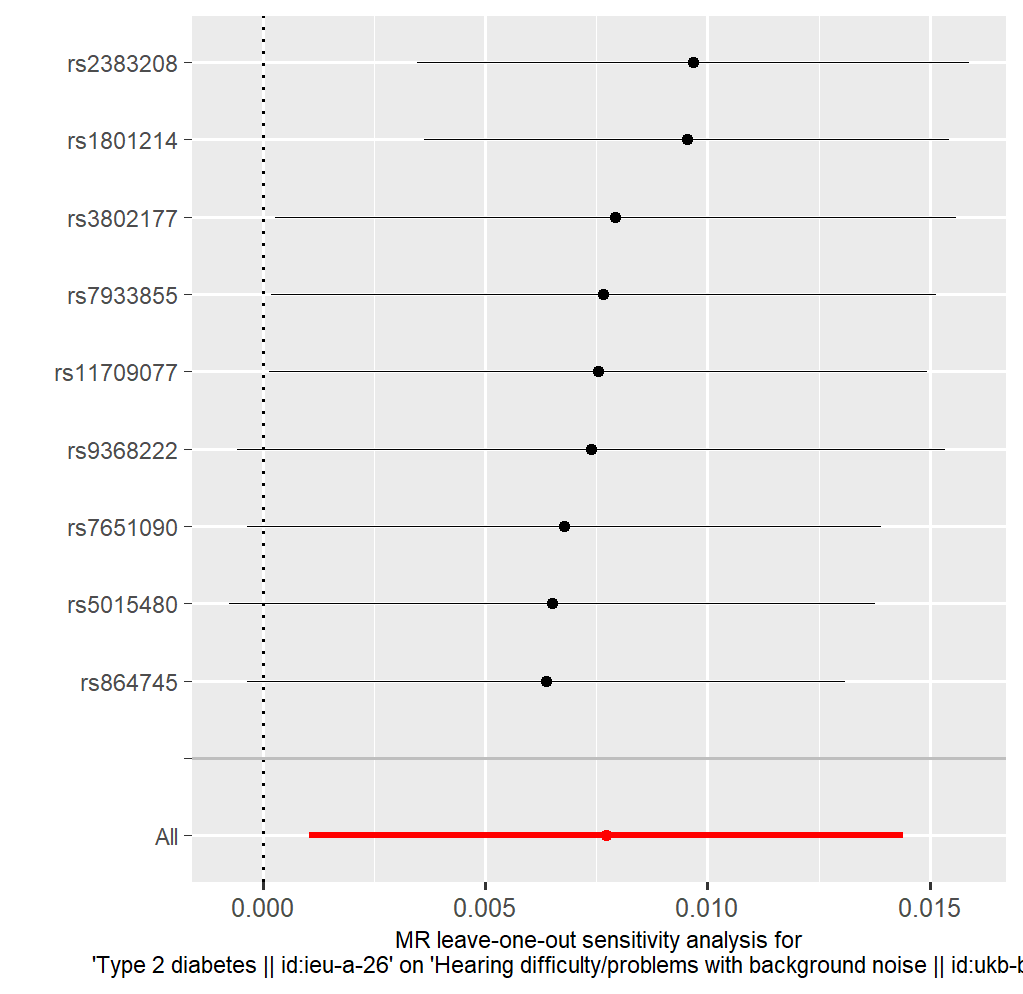


(G)


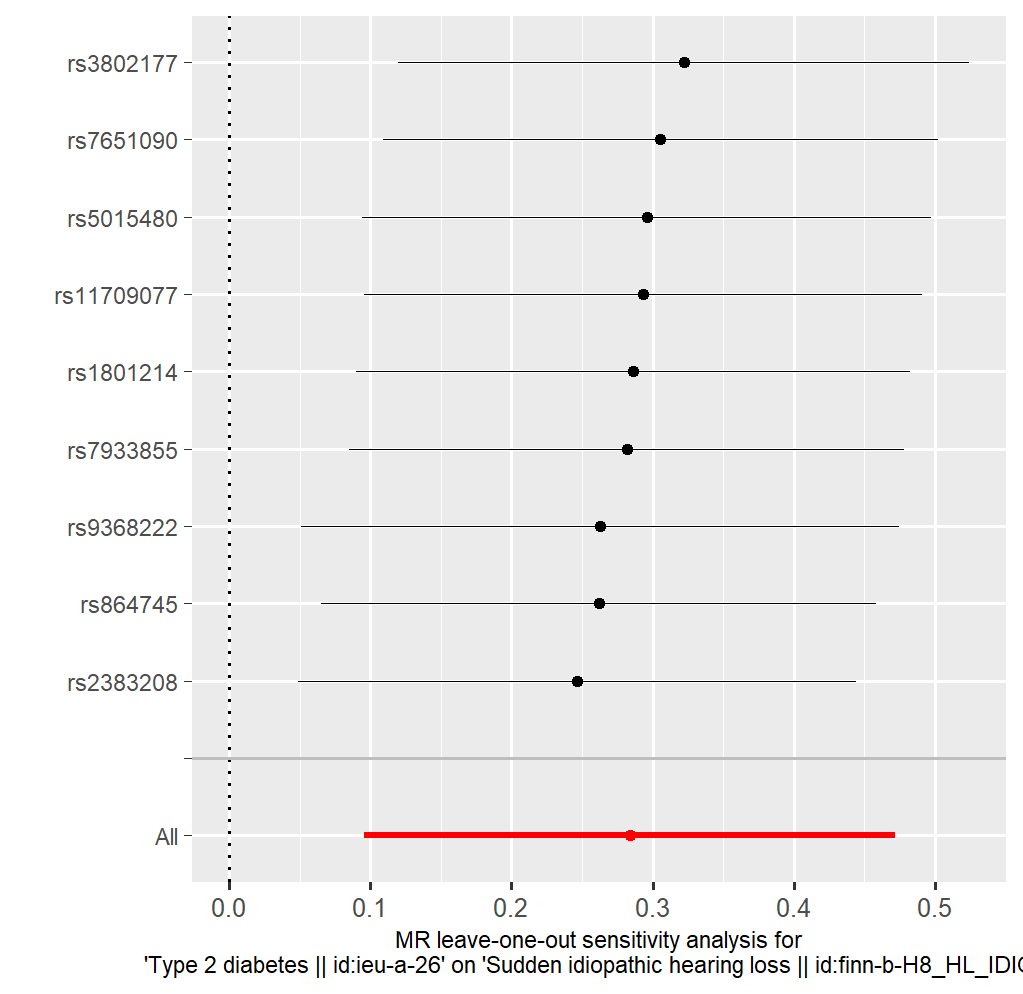


(H)


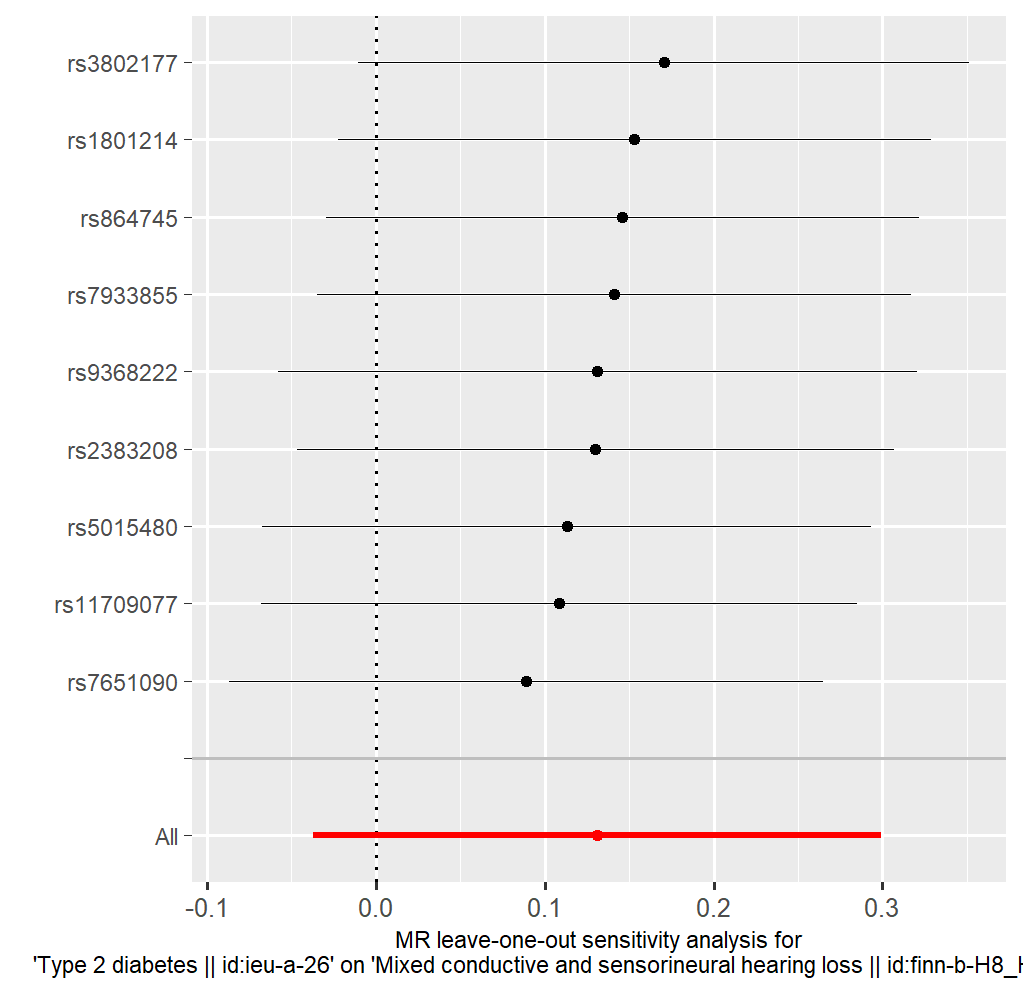


(I)


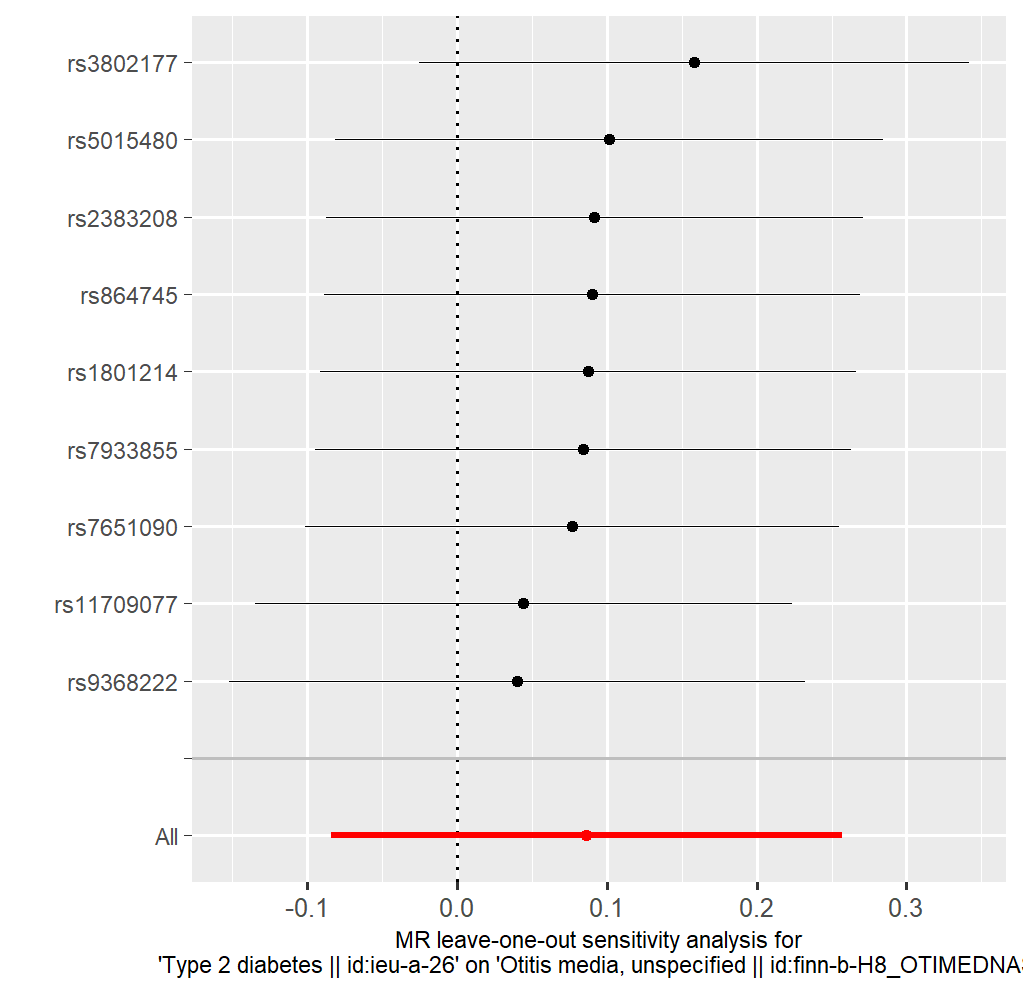


(J)
